# Malignant peripheral nerve sheath tumor (MPNST) and MPNST-like entities are defined by a specific DNA methylation profile in pediatric and juvenile population

**DOI:** 10.1186/s13148-023-01621-7

**Published:** 2024-01-04

**Authors:** Sara Patrizi, Evelina Miele, Lorenza Falcone, Silvia Vallese, Sabrina Rossi, Sabina Barresi, Isabella Giovannoni, Lucia Pedace, Claudia Nardini, Ilaria Masier, Luana Abballe, Antonella Cacchione, Ida Russo, Angela Di Giannatale, Valentina Di Ruscio, Claudia Maria Salgado, Angela Mastronuzzi, Andrea Ciolfi, Marco Tartaglia, Giuseppe Maria Milano, Franco Locatelli, Rita Alaggio

**Affiliations:** 1 Onco-Hematology, Cell Therapy, Gene Therapies and Hemopoietic Transplant, Bambino Gesù Children’s Hospital, IRCCS, Rome, Italy; 2 Pathology Unit, Bambino Gesù Children’s Hospital, IRCCS, Rome, Italy; 3 Division of Pathology, University of Pittsburgh Medical Center Children’s Hospital of Pittsburgh, Pittsburgh, PA USA; 4 Molecular Genetics and Functional Genomics, Bambino Gesù Children’s Hospital, IRCCS, 00146 Rome, Italy; 5 Present Address: Pathology Unit, Augusto Murri Hospital, Azienda Sanitaria Territoriale di Fermo – Marche, Fermo, Italy; 6 Department of Life Sciences and Public Health, Catholic University of the Sacred Heart, Rome, Italy

**Keywords:** Malignant peripheral nerve sheath tumors, DNA methylation, Sarcoma classifier, H3K27 trimethylation

## Abstract

**Background:**

Malignant peripheral nerve sheath tumors (MPNSTs) account for 3–10% of pediatric sarcomas, 50% of which occur in neurofibromatosis type 1 (NF1). Sporadic MPNSTs diagnosis may be challenging due to the absence of specific markers, apart from immunohistochemical H3K27me3 loss. DNA methylation (DNAm) profiling is a useful tool for brain and mesenchymal neoplasms categorization, and MPNSTs exhibit a specific DNAm signature. An MPNST-like group has recently been recognized, including pediatric tumors with retained H3K27me3 mark and clinical/histological features not yet well explored. This study aims to characterize the DNAm profile of pediatric/juvenile MPNSTs/MPNST-like entities and its diagnostic/prognostic relevance.

**Results:**

We studied 42 tumors from two groups. Group 1 included 32 tumors histologically diagnosed as atypical neurofibroma (ANF) (*N* = 5) or MPNST (*N* = 27); group 2 comprised 10 tumors classified as MPNST-like according to Heidelberg sarcoma classifier. We performed further immunohistochemical and molecular tests to reach an integrated diagnosis. In group 1, DNAm profiling was inconclusive for ANF; while, it confirmed the original diagnosis in 12/27 MPNSTs, all occurring in NF1 patients. Five/27 MPNSTs were classified as MPNST-like: Integrated diagnosis confirmed MPNST identity for 3 cases; while, the immunophenotype supported the change to high-grade undifferentiated spindle cell sarcoma in 2 samples. The remaining 10/27 MPNSTs variably classified as schwannoma, osteosarcoma, BCOR-altered sarcoma, rhabdomyosarcoma (RMS)-*MYOD1* mutant, RMS-like, and embryonal RMS or did not match with any defined entity. Molecular analysis and histologic review confirmed the diagnoses of BCOR, RMS-*MYOD1* mutant, DICER1-syndrome and ERMS. Group 2 samples included 5 high-grade undifferentiated sarcomas/MPNSTs and 5 low-grade mesenchymal neoplasms. Two high-grade and 4 low-grade lesions harbored tyrosine kinase (TRK) gene fusions.

By HDBSCAN clustering analysis of the whole cohort we identified two clusters mainly distinguished by H3K27me3 epigenetic signature. Exploring the copy number variation, high-grade tumors showed frequent chromosomal aberrations and *CDKN2A/B* loss significantly impacted on survival in the MPNSTs cohort.

**Conclusion:**

DNAm profiling is a useful tool in diagnostic work-up of MPNSTs. Its application in a retrospective series collected during pre-molecular era contributed to classify morphologic mimics. The methylation group MPNST-like is a ‘hybrid’ category in pediatrics including high-grade and low-grade tumors mainly characterized by TRK alterations.

**Supplementary Information:**

The online version contains supplementary material available at 10.1186/s13148-023-01621-7.

## Introduction

Malignant peripheral nerve sheath tumors (MPNST) are spindle cell sarcomas that frequently arise from peripheral nerve or pre-existing peripheral nerve sheath tumors. These tumors occur sporadically or, more commonly, are associated with neurofibromatosis type 1 (NF1) [1]. Because of their rarity before adulthood, very little is known about clinical management of MPNST in younger patients, though a highly aggressive clinical behavior has been reported in this age [2]. Outside the context of NF1, the diagnosis of MPNST may be challenging due to the absence of specific morphological, immunophenotypic, and molecular markers of Schwannian differentiation and the histologic overlap with other spindle cell malignancies [3].

The molecular pathogenesis of MPNST is still not completely known. Recent studies have identified loss-of-function mutations of Polycomb Repressive Complex 2 components EED and SUZ12, that lead to loss of histone H3 lysine 27 trimethylation (H3K27me3) [4]. Mutations of these epigenetic regulators cause MPNSTs to have a peculiar DNA methylation (DNAm) profile that can be a useful tool for diagnosis and clinical stratification of patients [5]. In addition, Röhrich et al. identified a separate MPNST group characterized by retained H3K27me3 and a distinctive DNAm profile. This group, termed “MPNST-like” is still under investigation, not being well defined from a clinical and histological point of view [6].

Here we evaluate the diagnostic usefulness of DNAm profiling for the classification of these tumors, and investigate the clinical characteristics of the MPNST-like group in a pediatric and juvenile STS cohort.

## Materials and methods

### Case selection

Between 2019 and 2022, we retrospectively analyzed all pediatric soft tissue tumors from patients followed at the Bambino Gesù Children’s Hospital in Rome, and pathology consults (RA). We selected cases with histologic diagnosis of MPNST (including malignant triton tumor, or MTT) or atypical neurofibroma (ANF) and/or MPNST/MPNST-like DNAm profile according to online platform DKFZ Sarcoma Classifier v12.2 (SC) [7] and retrieved their clinical information from clinical records.

## DNA Methylation profiling

DNAm analysis was carried out using the Human MethylationEPIC v1.0 BeadChip arrays (Illumina), according to the manufacturer’s instructions, as previously reported [8]. Raw methylation data were uploaded to the SC.

Raw BeadChip data were also analyzed with R package ChAMP (version 2.26.0) [9]. Data loading was performed with method “minfi”, followed by a filtering step that removed failed probes (with detection *p*-value > 0.01). Samples with a proportion of failed probes above 0.1 were excluded from the analysis. After functional normalization, Singular value decomposition (SVD) analysis was performed to identify possible confounding factors in the normalized dataset. Principal component analysis (PCA) was performed on the beta value of the 1000 most variable probes. Unsupervised clustering of PCA results was performed with Hierarchical density-based spatial clustering of applications with noise (HDBSCAN) as implemented in the dbscan R package v1.18 [10], setting the minimum cluster size parameter to 4.

To further characterize the differences among samples, we performed differential methylation analysis between the identified clusters using method Bumphunter with default parameters [11] as implemented in package ChAMP. We also looked for enriched genesets in the genes overlapping the significant differentially methylated regions (DMRs) using champ.GSEA with “fisher” method. Subsequently, we validated our observation by revision of diagnostic immunohistochemistry results.

## Histologic review

For each tumor, all available slides were critically reviewed after DNAm analysis. Additional immunohistochemistry was performed according to histology and to validate newly emerging diagnostic hypotheses, including H3K27me3, S100, SOX10, Desmin, MyoD1, Myogenin, and BCOR.

Immunohistochemical investigations were performed on 2 µm thick slices of formalin-fixed, paraffin-embedded tissue sections. The following antibodies were used on the Dako Omnis platform (Agilent): S100 (Dako Omnis pAbs ready-to-use, high pH), SOX10 (RabMAb Cell Marque mAb clone EP268 1:50, pH 7.2), tri-methyl histone H3K27me3 (Cell Signaling Technology mAb clone C36B11 1:200, low pH), and SALL4 (Biocare Medical mAb clone 6E3 1:150, high pH), following published guidelines. Immunohistochemical expression was quantitatively evaluated based on the percentage of tumor cells showing nuclear and cytoplasmic positivity for the S100 protein and only nuclear positivity for SOX10, SALL4, and H3K27me3. For H3K27me3, only cases with a clear positive internal control represented by lymphocytes and/or endothelium of blood vessels, were considered evaluable.

## Molecular analysis

When necessary, to further confirm the integrated diagnosis, reverse transcriptase polymerase chain reaction (RT-PCR) and parallel sequencing were performed on the available material. Parallel sequencing included RNA sequencing (RNA-seq), Archer FusionPlex custom panel, Archer VariantPlex custom panel, and Illumina TruSight Oncology 500 panel (TSO500) (details in Additional file 1: Supplementary methods).

## Copy number variation analysis

CNV analysis was performed by applying the segmentation algorithm implemented in R package conumee [12] to DNAm intensity data. For each sample, we generated genome-wide CNV plots to visually inspect abnormal number of copies of genomic segments, and computed the log_2_R value of 29 cancer-related genes, that are usually highlighted in the CNV plots generated by the SC [13]. Changes in the copy number were considered when mean log_2_R was less than  − 0.2 (loss) and above 0.2 (gain). We further characterized the main histological groups, including MPNST samples with MPNST DNAm profile, ANFs, high-grade and low-grade sarcomas with MPNST-like DNAm profile, through cumulative CNV plots generated with R package GenVisR [14].

## Survival analysis

Kaplan–Meier survival curves were calculated using R package survival [15], according to histological diagnosis and *CDKN2A/B* loss status.

## Results

We identified 42 tumors from 37 patients from two groups. Group 1 included 32 samples with the following histologic diagnoses: ANF (*N* = 5), MPNST (*N* = 27, of which 4 MTT); while, group 2 comprised 10 cases with various histologic diagnosis classified as MPNST-like according to DNAm profile analysis. For 4 patients, we analyzed multiple samples: the primary (case #1) and metastatic (case #8) lesions of one patient (patient 1), and metachronous nerve sheaths lesions of 3 NF1 patients (case #18 and #19; #7 and #24; #5, #23 and #29). The age range was 0.2–24 years (median: 12 years); 18 patients were females and 19 males.

**Fig. 1:**
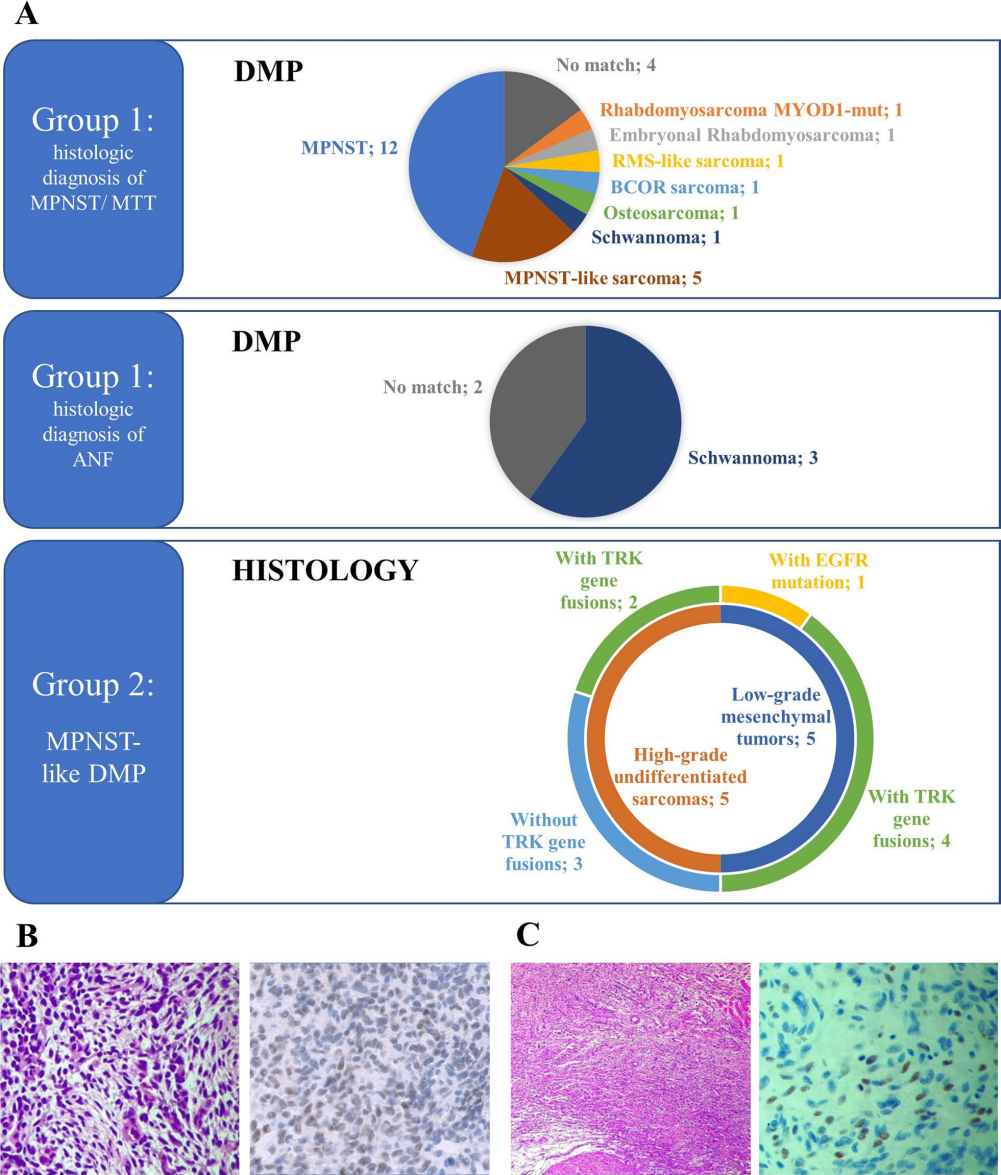
Sarcoma classifier results and histologic review of selected cases. **A**: Results of group comparisons based on DNAm profiling analysis with Sarcoma classifier. Group 1 comprises tumors with homogenous histology, malignant peripheral nerve sheath tumors (MPNST)/malignant triton tumor (MTT)—upper panel, and atypical neurofibroma (ANF)—lower panel, so the pie charts represent the different results of DNAm profiling. Conversely, samples of group 2 were selected because of a homogenous MPNST-like DNAm signature, and the donut chart represents the intra-group histological variability. **B**: Hematoxylin–eosin staining (left) and immunohistochemistry results for BCOR (right) confirming the diagnosis of BCOR-altered sarcoma suggested by DNAm profiling for case #11. **C**: Hematoxylin–eosin staining (left) and immunohistochemistry results for MyoD1 (right) confirming the diagnosis of RMS *MYOD1-*mutated suggested by DNAm profiling for case #12. Picture magnification is 20X (B and C, right panel) or 4X (C, left panel)

The results of group comparisons based on DNAm profile analysis with SC are detailed below and represented in Fig. 1A and Table 1.

**Table 1:** Demographic, clinical and diagnosis details of the sample cohorts

Case (#)	Gender	Age at diagnosis (years)	Location	Initial histological diagnosis	Integrated diagnosis
*Group 1*					
1	M	9	left lower limb	MPNST	HGUSCS
2	M	22	paraspinal	MTT	DICER1 syndrome-associated sarcoma
3	M	12	torax	MTT	MTT in NF1
4	M	15	torax	MPNST	MPNST
5	M	16	head and neck	MPNST	MPNST
6	F	17	upper limb	ANF	ANF
7	F	18	left lower limb	MPNST	MPNST
8	M	9	lung metastasis	MPNST	HGUSCS
9	F	NA	NA	MPNST	MPNST
10	M	NA	NA	MPNST	MPNST
11	F	0.17	pelvis	MPNST	*BCOR*-altered sarcoma
12	F	3	lower limb	MTT	Spindle cell RMS, *MYOD1*-mut
13	M	18	torax	MPNST	MPNST
14	M	12	head and neck	MPNST	MPNST
15	M	NA	NA	MPNST	MPNST
16	F	NA	NA	MPNST	MPNST
17	M	NA	NA	MPNST	MPNST
18	F	19	left lower limb	MPNST	MPNST
19	F	18	left lower limb	MPNST	MPNST
20	F	16	abdomen	MPNST	MPNST
21	M	24	torax	MPNST	MPNST
22	F	19	left upper limb	MPNST	MPNST
23	M	17	right iliac fossa	MPNST	MPNST
24	F	19	left lower limb	MPNST	MPNST
25	M	10	torax	MPNST	MPNST
26	M	13	NA	MPNST	MPNST
27	F	2	pelvis	MTT/RMS	RMS in NF1
28	M	10	right upper limb	ANF	ANF
29	M	14	head and neck	MPNST	MPNST
30	F	15	head and neck	ANF	ANF
31	F	11	left upper limb	ANF	ANF
32	M	20	left upper limb	ANF	ANF
*Group 2*					
33	M	11	left lower limb	HGUSCS	HGUSCS
34	F	4	retroperitoneum	HGUSCS	HGUSCS
35	F	3	kidney	IF	Mesenchymal *TPM3::NTRK1* rearranged neoplasia (low-grade)
36	M	2	right arm	IF	Mesenchymal low-grade myofibroblastic neoplasia
37	M	1	kidney	HGUSCS/cellular mesoblastic nephroma	Mesenchymal *EML4::NTRK3* rearranged neoplasia (high grade)
38	F	2	NA	IF	Mesenchymal *CLIP::RAF1* rearranged neoplasia (low-grade)
39	F	0.25	head and neck (tongue)	Infantile TRK-rearranged mesenchymal tumor	Spindle cell mesenchymal *RPBMS::NTRK3* rearranged neoplasia
40	F	12	arm	HGUSCS	High-grade undifferentiated pleomorphic sarcoma
41	F	5	upper limb	TRK-rearranged mesenchymal tumor	*TRK*-rearranged mesenchymal tumor
42	M	2	kidney	High-grade infantile TRK-rearranged mesenchymal tumor	Pediatric *NTRK*-rearranged spindle cell neoplasm

MPNST: Malignant peripheral nerve sheet tumor; MTT: Malignant triton tumor; ANF: atypical neurofibroma; RMS: Rhabdomyosarcoma; NF1: Neurofibromatosis type 1; HGUSCS: High-grade undifferentiated spindle cell sarcoma; IF: infantile fibrosarcoma; NA: not available

Group 1: Among the 5 atypical neurofibromas, all arising in NF1 patients, 3 classified as schwannoma and 2 did not match any entity. The integrated diagnosis confirmed the initial histologic findings.

In 12 out of 27 MPNSTs, all occurring in NF1 patients, the initial histologic diagnosis was confirmed by DNAm profile analysis. Five out of 27 cases were classified in the MPNST-like category, including 1 MTT in NF1, 2 MPNSTs in NF1, and 2 sporadic MPNSTs. Three cases were further molecularly characterized, respectively by RT-PCR for the *SYT*::*SSX*, *ETV6*::*NTRK3*, *TPM3*::*NTRK1* rearrangement (*n* = 1), and by Archer FusionPlex (*n* = 2), finding no fusions. According to immunohistochemical findings, the final diagnosis was changed to “high-grade undifferentiated spindle cell sarcoma” for two cases belonging to the same patient, since both displayed positive H3K27me3, and one was also negative for SOX10. In addition, no relationship with nerve trunk was observed.

The remaining 10/27 MPNSTs were classified in the schwannoma (1 sporadic MPNST), osteosarcoma high grade (1 MPNST in NF1), *BCOR*-rearranged sarcoma (1 sporadic MPNST), RMS-*MYOD1*mut (1 sporadic MTT), RMS-like (1 MTT in NF1), and ERMS category (1 MTT in NF1), or did not match any category (4 sporadic MPNSTs). The match in unexpected methylation classes prompted further analyses. The initial diagnosis of MPNST was confirmed for the “no-match” cases, and those matching as schwannoma or osteosarcoma: the “no-match” cases were indeed old and low-quality samples, the schwannoma matching case had a low confidence score, and the latter, despite the high calibrated score in the osteosarcoma class, clustered with the other MPNSTs in our internal pipeline (data not shown). Immunohistochemistry for BCOR (Fig. 1B) and MYOD1 (Fig. 1C) were positive in the two cases classified as *BCOR*-rearranged sarcoma and *MYOD1-*mutant RMS, respectively (#11 and #12), thus corroborating the diagnoses suggested by DNAm profiling. RT-PCR for *BCOR* rearrangements or DNA sequencing for exon 1 mutations of *MYOD1* failed due to poor nucleic acid quality in both cases. DICER syndrome, which was suggested by the RMS-like DNAm profile (case #2), was confirmed by parallel sequencing [16]. One case classified as ERMS by DNAm profile (case #27) showed histologic appearance of rhabdomyoblastic differentiation, which had been interpreted in the context of a diagnosis of MTT, in line with previous observations [17]. Based on DNAm profiling, the case was critically revised, and the final integrated diagnosis was changed to ERMS.

In group 2, the 10 tested sarcomas showed an MPNST-like DNAm signature. This heterogeneous category included 5 high-grade sarcomas with a morphology shared by both MPNST and undifferentiated sarcoma. Immunohistochemically H3K27me3 was preserved in all cases; while, S100 staining was negative in 3/5 cases. Tyrosine kinase (*TRK*) gene fusions were identified in 2/5 cases. Another group of MPNST-like cases included 5 low-grade mesenchymal neoplasms, of which 4 with *TRK* gene fusions (detected by either Archer VariantPlex or RNA-seq) and 1 with *EGFR* mutation (found by RNA-seq).

**Fig. 2:**
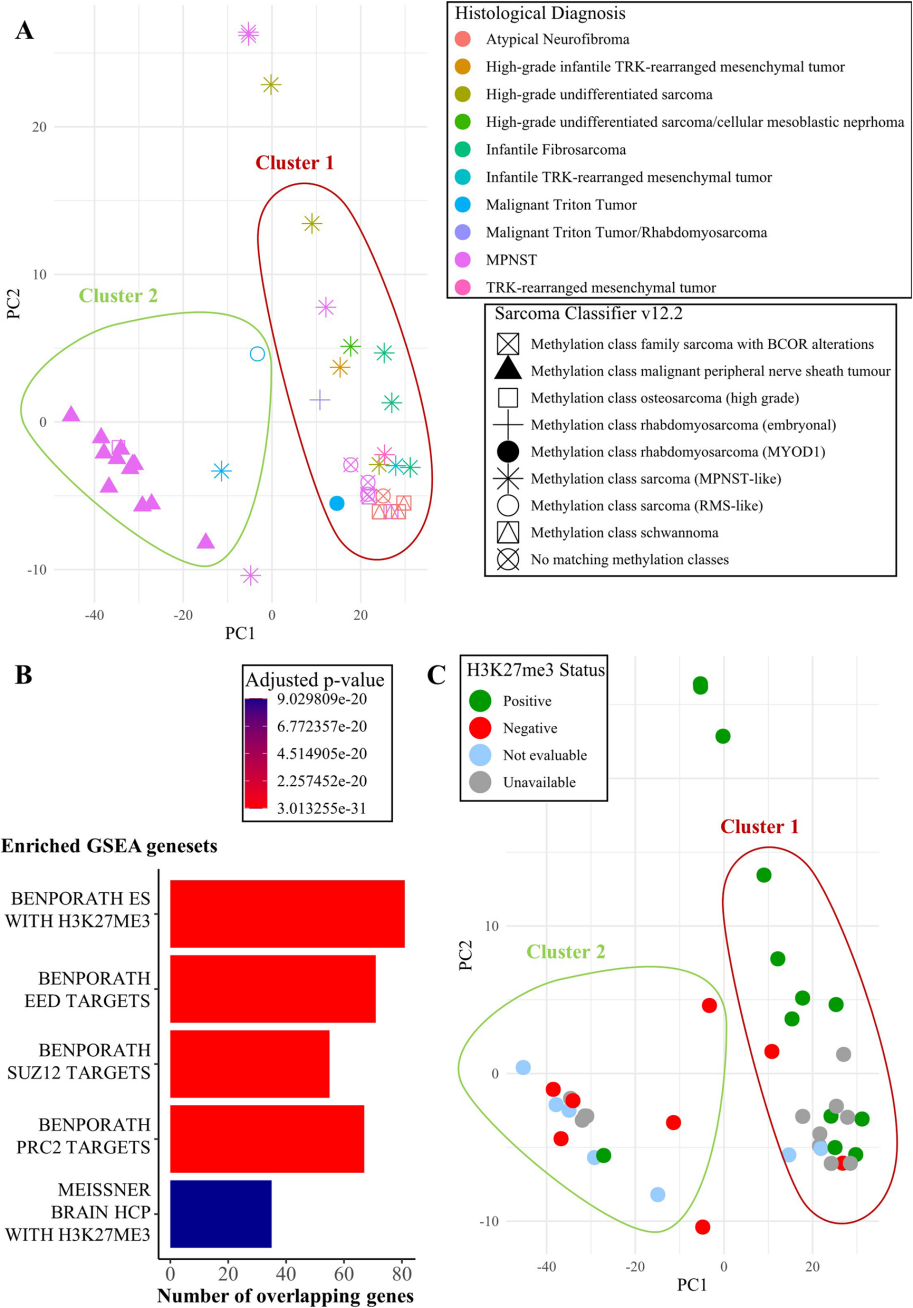
DNA methylation analysis results. A: PCA clustering of sarcoma DNAm profiling. Histological diagnosis is represented by point color, DNAm profiling results according to the Sarcoma Classifier are represented by point shape; clusters are indicated by green or red margins. B: Barplot of top 5 GSEA results for the differentially methylated genes between cluster 1 and cluster 2. For each enriched geneset (Y axis), bar length (X axis) represents the number of differentially methylated genes included in the geneset, and bar color represents the adjusted p-value. C: PCA clustering results where H3K27me3 status is represented by sample color

To further understand the biological significance of our results, we performed additional analyses on DNAm data. First, we carried out principal component analysis (PCA) on methylation beta values of the 1000 most variable probes across 40 samples. One Atypical neurofibroma case (#10) and one MPNST case (#28) were excluded due to poor quality. The excluded samples also did not match to any methylation class according to the SC. HDBSCAN clustering applied to PCA results assigned 21 samples to cluster A, 15 samples to cluster B, and identified 4 samples as outliers. In cluster A, we observed a prevalence of MPNST-like DNAm profile (10/21), though 11 samples matching other methylation classes (BCOR sarcoma, ERMS, RMS-MYOD1mut, Schwannoma, or no match) also mapped within this cluster. Cluster B was more homogenous and mostly included samples with MPNST DNAm profile (12/15) (Fig. 2A).

Of note, 3 samples were assigned to cluster B despite what was expected based on their classifier result: 1 MTT in NF1, that classified as MPNST-like, 1 MPNST in NF1 that classified as osteosarcoma, and 1 MTT in NF1 that classified as RMS-like.

We compared samples in cluster A with samples in cluster B obtaining 340 significantly differentially methylated regions, that overlapped 327 genes. The geneset “BENPORATH_ES_WITH_H3K27ME3” was the most significantly enriched among the differentially methylated genes (Fig. 2B). This geneset was identified with a ChIP-on-chip approach by Ben-Porath et al. as the genes possessing H3K27me3 mark in their promoters in human embryonic stem cells [18].

To confirm the differential H3K27me3 levels between clusters, we reviewed the immunohistochemistry data for H3K27me3. Overall, this marker was at least partially retained (positive) in 14/42 cases and lost (negative) in 8/42. Its expression was not evaluable in 7/42 cases, and analysis was not possible in 13/42 due to unavailable tissue samples. Most positive cases (9/14) were in cluster A and most negative cases (5/8) in cluster B (Fig. 2C).

**Fig. 3:**
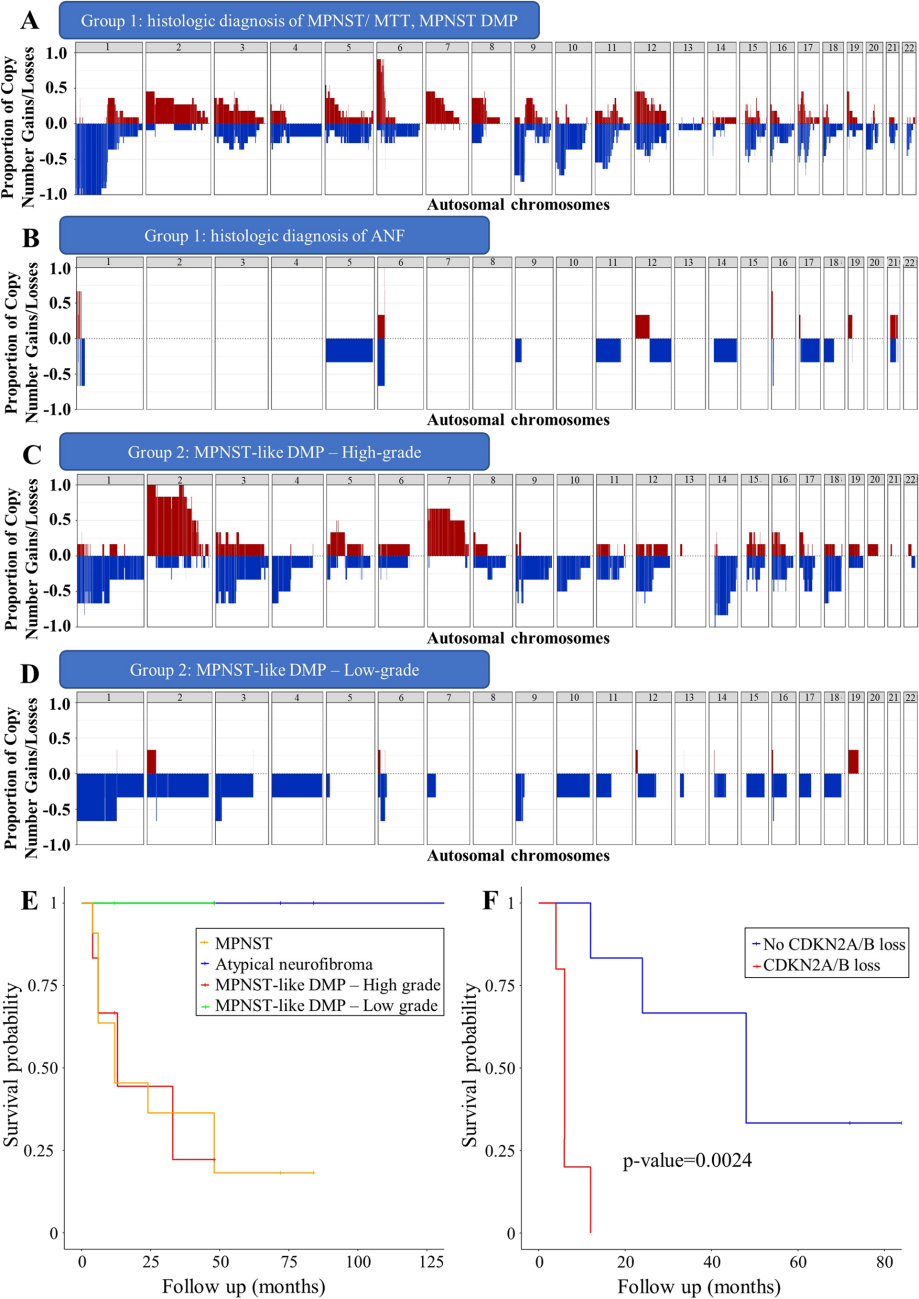
CNV and survival analysis results. **A-D:** Cumulative CNV plots representing the proportion of copy number gains (red) or losses (blue) across all chromosomes in MPNST samples of group 1 **A**, ANF samples **B**, high-grade **C** and low-grade **D** sarcomas with MPNST-like DNAm profile. **E:** Kaplan–Meier survival curve for the different histological types included in the study (MPNST in yellow, atypical neurofibroma in blue, MPNST-like DNAm profile – High grade in red, MPNST-like DNAm profile – Low grade in green. **F:** Kaplan–Meier curve representing the effect of CDK2A/B loss (absent in blue, present in red) on the survival of patients with MPNST histology and MPNST DNAm profile

Then, we focused on CNV data looking for recurrent or prognostic alterations. Upon visual inspection, the cohort was highly variable: 29 samples, including high-grade sarcomas and MPNSTs/MTTs, displayed a complex profile with multiple abnormal number of copies; 13 samples had a low quantity of alterations, and were mainly ANFs/low-grade sarcomas (Additional file 2: Fig. S1 and Additional file 3: Table S1). Notably, MPNSTs were characterized by gains and/or losses involving every chromosome (Fig. 3A); while, the ANF samples had a predominantly flat profile (Fig. 3B).

Among MPNST-like samples in Group 2, the high-grade undifferentiated sarcomas were characterized by the presence of frequent chromosomal aberrations and genetic alterations, such as loss of *CDKN2A/B* on chromosome 9p and *C19MC* on chromosome 19q (Fig. 3C and Additional file 4: Table S2); while, the morphologically and clinically low-grade mesenchymal neoplasms with tyrosine kinase gene fusion had minimal or absent CNVs (Fig. 3D).

Follow-up data were available for 32/42 cases. At the time of data collection, 14 patients were alive (7 in the NF1 group), 18 died of disease (13 in the NF1 group); whereas, 10 (all consults) were lost at follow-up. Through Kaplan–Meier analysis, we observed that MPNSTs, ANF, low-grade and high-grade MPNST-like samples had different survival rates, although the differences were not statistically significant (Fig. 3E). *CDKN2A/B* loss negatively impacted survival in the entire cohort, albeit not in a statistically significant manner (Additional file 2: Fig. S2). Among the MPNST cases, the effect of *CDKN2A/B* loss on survival was statistically significant (*p*-value = 0.0024, Fig. 3F).

## Discussion

Our study reveals an unexpected molecular heterogeneity in apparently histologically homogenous MPNSTs, and histological diversity in samples with MPNST-like profiles. We confirm the rarity of MPNST in pediatric population outside the context of NF1, as demonstrated by a reclassification of 6 out of 27 cases with initial diagnosis of MPNST or MTT. The latter group shows a particularly variable DNAm profile, since they comprise 4 out of the 6 reclassified cases. Our findings are in line with a previous report outlining the challenging diagnosis and categorization of these entities in the pediatric age group [19].

Being this a retrospective study, it also reflects the lack of immunohistochemical markers not yet available at time of diagnosis. However, the collected data also demonstrate the valuable role of DNAm profiling in the diagnostic work-up of MPNSTs in childhood and young adulthood.

A *MYOD1*-mutated RMS had been originally diagnosed as MTT. Although the occurrence of a *MYOD1* mutation could not be demonstrated by molecular characterization, the extensive positive immunostaining for MyoD1 and myogenin, which was not performed at the time of the first evaluation, is supportive for the diagnosis. Similarly, the case classified as BCOR family sarcoma based on DNAm profiling (case 11) was antecedent to the identification of this category, but critical re-evaluation of the tumor confirmed the histologic appearance characterized by typical curvilinear vascular pattern, elongated cells with nuclei showing homogeneous finely dispersed chromatin, and a myxoid background, which is consistent with the diagnosis and positive nuclear immunostaining for BCOR. This finding is in line with other studies proving that in the past some *BCOR*-altered malignancies (*ITD* and *CCNB3*-fused) were misdiagnosed as MPNSTs [20]. Although molecular confirmation of the *MYOD1* and *BCOR* alterations failed, DNAm profiling showed 100% sensitivity and specificity in these cases.

DICER1 sarcoma (case 2) was originally diagnosed as MTT based on the loss of H3K27me3 expression on immunohistochemistry. However, the DNAm profile of this tumor classified it as RMS-like, a family that is characterized by *DICER1* alterations. Moreover, immunohistochemistry was positive for stemness marker SALL4, which is typically associated with loss of H3K27me3 in DICER1 sarcomas. The evidence of biallelic inactivation of the gene detected in the neoplastic tissue by parallel sequencing prompted genetic analysis on blood, leading to the identification of DICER1 syndrome in our patient [16].

In a patient affected with NF1 (Case #27), complete loss of H3K27me3 with focal expression of S100 and myogenic markers led to the diagnosis of MTT. Despite the predominant histologic appearance of ERMS, our finding is in agreement with the recent study by Hornich et al. [17] suggesting that H3k27me3 loss is supportive of a MPNST even in presence of a diffuse rhabdomyosarcomatous appearance. DNAm profiling classified this tumor as ERMS, which is in contrast with this hypothesis and supports the possibility that ERMS may arise in the context of NF1 [21]. Consistently, loss of expression of H3k27me3 has been reported in ERMS [22].

From one patient, who was not affected with NF1, we collected both the primary and metastatic neoplasia that were initially diagnosed as MPNSTs. Match of both tumors to the MPNST-like class, together with the retained H3K27me3 in both samples, and the negativity for SOX10 of the primary lesion, led to a final diagnosis of high-grade undifferentiated spindle cell sarcoma.

The differential diagnosis of MPNST from atypical neurofibroma (ANF)/ atypical neurofibromatous neoplasm of uncertain biological potential (ANNUBP) typical of NF1 may be challenging and is based on the presence of at least two features among cytological atypia, hypercellularity, loss of neurofibroma architecture and mitotic count [23]. Our cases displayed all these features, hence the ANF histological diagnosis. Since the specific category of ANF does not exist as a methylation class in the SC, 3 out of 5 samples that had a reliable match fell into the most closely related category of schwannoma. This DNAm profile similarity reflects the histological closeness of this tumor family [24, 25]. All patients with available follow-up were alive (4/5), corroborating the diagnosis.

In total, 15 out of 42 samples showed an MPNST-like DNAm profile. Five belonged to study group 1, and in three of those cases the original diagnosis of MPNST/MTT was maintained; while, the DNAm profile prompted further analyses in 2 cases, that were subsequently reclassified as high-grade undifferentiated sarcomas. Study group 2 included 10 cases, divided into two different groups. The first group comprised high-grade undifferentiated sarcomas (5 cases) showing frequent segmental alterations and gene gains/losses, two of them with tyrosine kinase gene fusions. In the second group, 5 morphologically and clinically low-grade mesenchymal neoplasms showed tyrosine kinase gene fusions/*EGFR* mutations and minimal or absent CNVs. Most patients affected by MPNST or high-grade sarcomas that classified as MPNST-like were dead of disease at last follow-up. This supports the notion that the prognosis can be assessed by studying tumor morphology and CNVs [8]. Of note, *CDKN2A/B* loss significantly correlated with worse survival in canonical MPNSTs confirming its prognostic value [26]. *CDKN2A*/p16 inactivation is a well-recognized key event in the malignant transformation of neurofibromas in NF1 [27].

Overall, the MPNST-like category does not identify a homogeneous group of lesions and should be evaluated with adequate morphological integration and molecular methods (NGS), since it may include lesions with genetic alterations that could be targeted by specific drugs. The small number of cases, due to their rarity, does not allow further correlations with prognosis, and CNV analysis.

A wider methylome analysis provided further insights into the variability of the study cohort. We identified two very distinct clusters, one with a prevalence of MPNST and one with a prevalence of MPNST-like methylation profile. The most enriched pathway among the genes that were differentially methylated between the two clusters was “BENPORATH_ES_WITH_H3K27ME3”, pointing to an already investigated relationship between the well-known epigenetic marker of MPNSTs, which is the loss of H3K27me3, and DNA methylation [28]. In our small-scale study, we could only differentiate two clusters among which H3K27me3 presence/loss influenced the main DNAm profile differences. The Sarcoma Classifier, however, has the capacity to differentiate several malignancies from each other regardless of H3K27me3 status, thanks to its machine learning algorithm that was developed using a large training cohort [7].

## Conclusions

In conclusion, our study emphasizes the usefulness of DNAm profiling in pediatric/juvenile MPNSTs. We analyzed a series of pediatric and young adult neoplasms with a histological diagnosis of MPNST or ANF and/or an MPNST or MPNST-like methylation profile. Tumors with an MPNST-like methylation profile represent a heterogeneous category, including high-grade malignant spindle cell sarcomas, better classified as undifferentiated sarcomas according to the WHO classification, and low-grade lesions with tyrosine kinase gene fusion. Soft tissue sarcomas associated with *TRK-* and other kinase fusions are an expanding group of neoplasm [29, 30] that have been recently recognized and characterized also in NF1 syndrome [31]. Such entities deserve further investigations and specific attention, also in light of the therapeutic targets. Within this group, the methylation profile in our hands had strong limitations and implementation with other methods is necessary. Conversely, methylation profiles have been useful in identifying undifferentiated sarcomas with specific transcripts or mutations, such as *BCOR-*altered undifferentiated sarcomas. The availability of an extensive immunostaining panel aids in the diagnosis of spindle cell rhabdomyosarcoma and DICER1 syndrome-associated sarcomas. However, DNAm profiling could help in those cases where the differential diagnosis from malignant triton tumors can be challenging, especially in small biopsies that may preclude the important identification of myogenic differentiation.

### Supplementary Information

**Additional file 1:** Supplementary methods.

**Additional file 2:** **Supplementary Figure 1.** Genome-wide CNV plots generated with conumee for each corresponding case number (#1-#42). Log2Ratio is represented on the Y axis, while the X axis represents the chromosomal position.** Supplementary Figure 2.** Kaplan-Meier curve representing the effect of CDKN2A/B loss (absent in blue, present in red) on all patients’ survival.

**Additional file 3: Supplementary Table 1.** Clinico-pathological and molecular information of the analyzed patients cohort.

**Additional file 4: Supplementary Table 2.** List of gain/loss status of 29 cancer related genes in the patients’ cohort.

## Data Availability

The datasets generated and/or analyzed during the current study are deposited in NCBI’s Gene Expression Omnibus, and accessible through the following link: https://www.ncbi.nlm.nih.gov/geo/query/acc.cgi?acc=GSE246644
