# Supplementary material for: Malignant peripheral nerve sheath tumor (MPNST) and MPNST-like entities are defined by a specific DNA methylation profile in pediatric and juvenile population
Source: Clin Epigenetics. 2024 Jan 4;16:9. doi: 10.1186/s13148-023-01621-7 (PMC10768529; doi:10.1186/s13148-023-01621-7)
Supplement: Supplementary file 1 — Additional file 1: Supplementary methods. [file 13148_2023_1621_MOESM1_ESM.docx]

**Supplementary Methods**

Nucleic acids extraction was performed from 5-10 unstained sections per sample from paraffin blocks. Significant areas containing ≥ 70% tumor cell, based on hematoxylin and eosin stained slides review, were scraped to perform extraction of DNA (by MagPurix FFPE DNA Extraction Kit, Zzinexts, Life Science Corporation, New Taipei City, Taiwan) and RNA (by ReliaPrep™ FFPE Total RNA kit, Promega).

The nucleic acid concentrations were measured on a Qubit 2.0 Fluorometer (Thermofisher Scientific, Waltham, USA) using the Qubit dsDNA and RNA High Sensitivity kit.

Three-hundreds nanograms of total RNA were utilized for the preparation of an NGS library using the SureSelect XT HS2 kit (Agilent Technologies), following the manufacturer's guidelines. The raw reads underwent preprocessing using Fastp, to eliminate low-quality reads and adapter sequences. Reads were aligned to the reference human genome (UCSC-Build38) with the STAR (v.2.5.3a) algorithm developed by Chen et al. in 2018. The resulting alignment files were then screened for potential fusion transcripts utilizing the Arriba and FusionCatcher pipelines, as described by Uhring et al. in 2021 and Nicorici et al. in 2018.

Two hundred nanograms of RNA and DNA were used for library preparation with the Archer Custom Fusion Plex Kit (Integrated DNA Technologies, IA) and Archer Custom Variant Plex Kit (Integrated DNA Technologies, IA) respectively, according to the manufacturer's protocols. RNA libraries were pooled in equimolar amounts of 4 nM (12 libraries/pool) and loaded at 10 pM with MiSeq Reagent Kits v3 600 cycles on MiSEQ platform (Illumina, San Diego, California). DNA libraries were pooled in equimolar amounts of 4 nM (20 libraries/pool) and loaded at 1.2 pM using NEXTSEQ 550 platform (Illumina, San Diego, California). The sequencing run was performed in paired-end mode (2 X 151-bp reads) using the Illumina MiSeQ platform. NGS data were analysed using Archer Data Analysis Software v6.2.3.

We also performed TruSight Oncology 500 (TSO500) assay (Illumina, San Diego, USA), a commercial NGS panel targeting 523 cancer-relevant genes. DNA libraries, obtained through the TSO500 Library Preparation Kit (Illumina, San Diego, USA) were pooled and denatured following the manufacturer’s protocol, then sequenced using NEXTSEQ 550 platform (Illumina, San Diego, USA) in paired-end mode (2 X 101-bp reads) with a mean coverage depth above 500X. Raw sequence data was analyzed with Illumina TruSight Oncology 500 Local App v2.1 and Pierian Clinical Genomics Workspace cloud (Pierian DX software CGW_V6.21.1).
