# Supplementary material for: Malignant peripheral nerve sheath tumor (MPNST) and MPNST-like entities are defined by a specific DNA methylation profile in pediatric and juvenile population
Source: Clin Epigenetics. 2024 Jan 4;16:9. doi: 10.1186/s13148-023-01621-7 (PMC10768529; doi:10.1186/s13148-023-01621-7)

Supplementary figure legends

Supplementary Figure 1: Genome-wide CNV plots generated with conumee for each corresponding case number (#1-#42). Log2Ratio is represented on the Y axis, while the X axis represents the chromosomal position.

Supplementary Figure 2: Kaplan-Meier curve representing the effect of CDK2A/B loss (absent in blue, present in red) on all patients' survival.

Supplementary Figure 1

#1

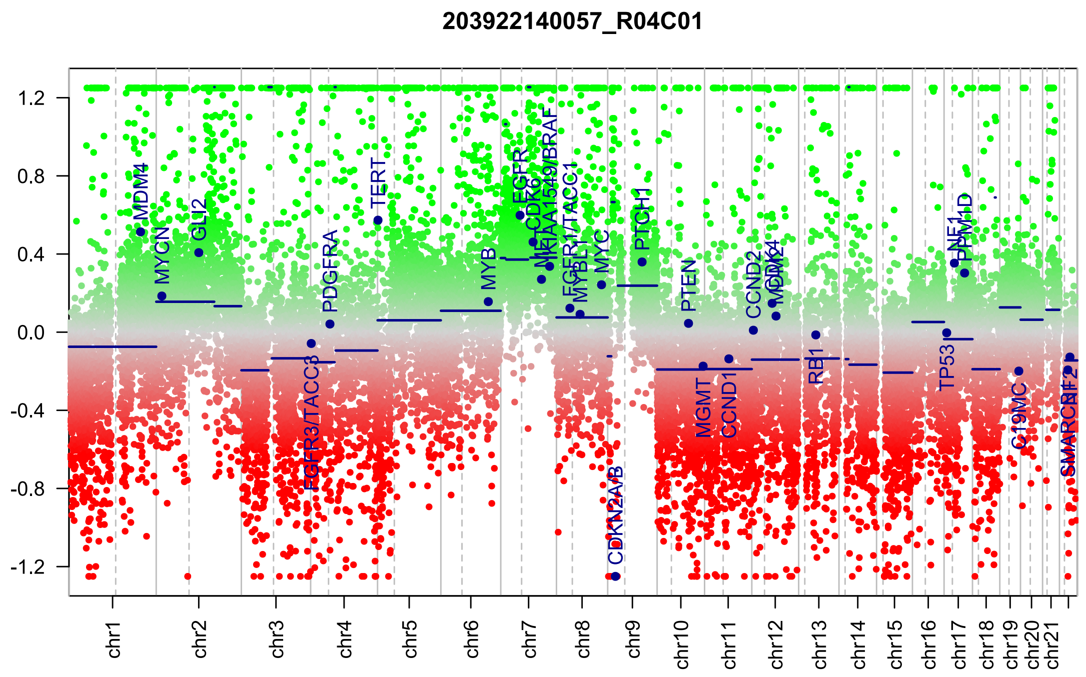

#2

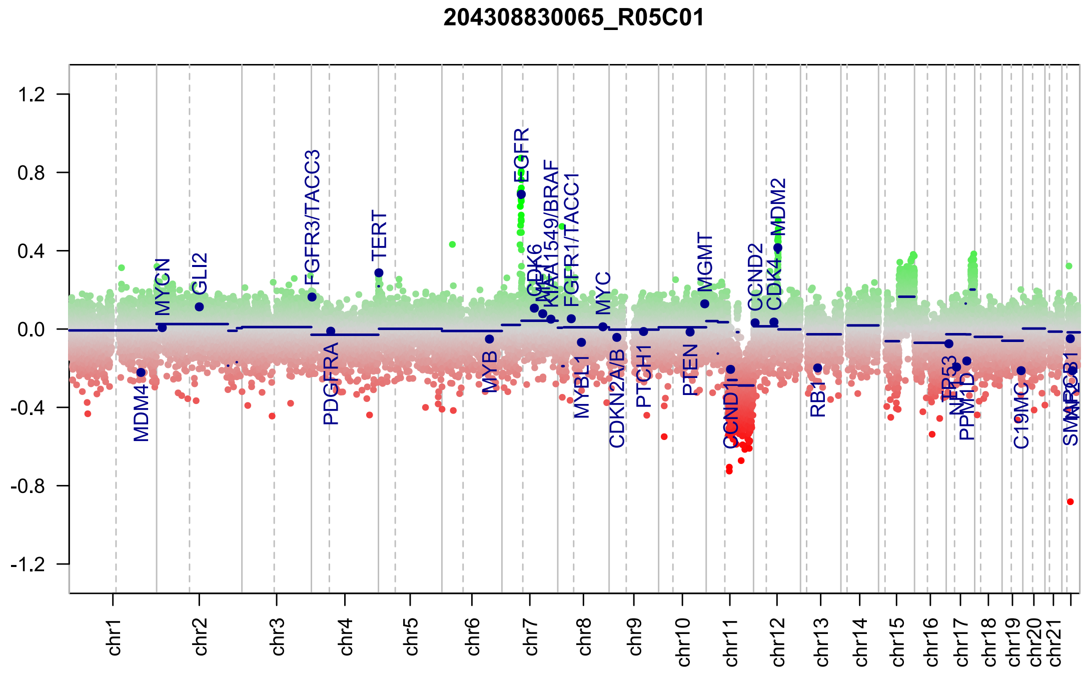
204308830065_R05C01
1.2
0.8
0.4
0.0
-0.4
-0.8
-1.2
chr1
chr2
chr3
chr4
chr5
chr6
chr7
chr8
chr9
chr10
chr11
chr12
chr13
chr14
chr15
chr16
chr17
chr18
chr19
chr20
chr21
MDM4
MYCN
GLI2
FGFR3/TACC3
PDGFRA
TERT
MYB
EGFR
CDK6
MET
KIAA1549/BRAF
FGFR1/TACC1
MYBL1
MYC
CDKN2A/B
PTCH1
PTEN
MGMT
CCND1
CCND2
CDK4
MDM2
RB1
TP53
NF1
PPM1D
C19MC
SMARCB1

#3

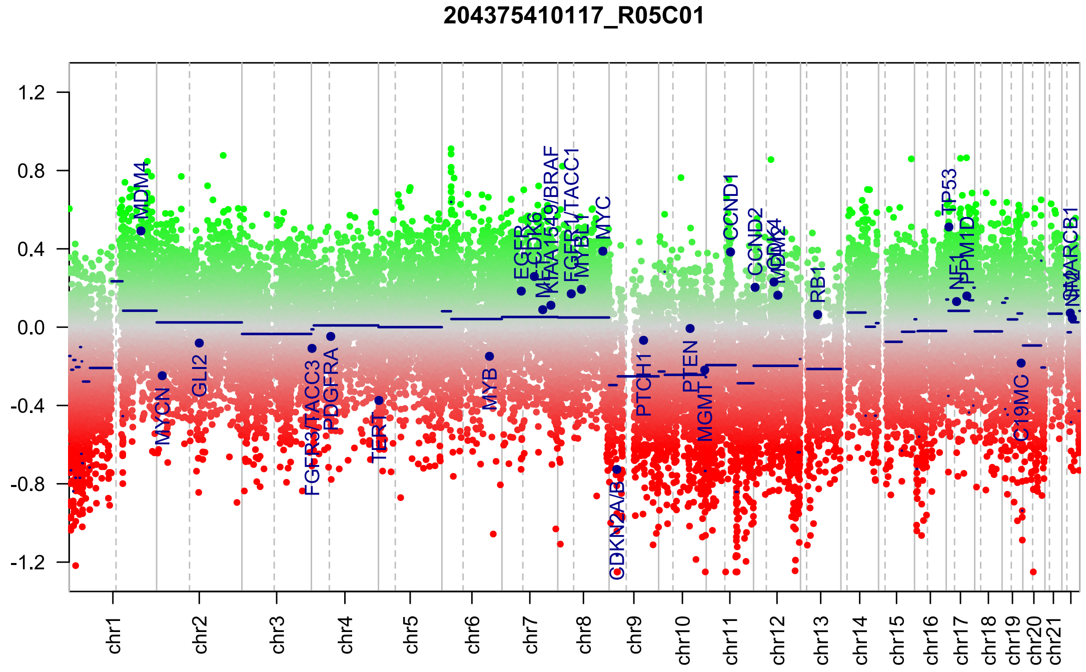
204375410117_R05C01
1.2
0.8
0.4
0.0
-0.4
-0.8
-1.2
chr1
chr2
chr3
chr4
chr5
chr6
chr7
chr8
chr9
chr10
chr11
chr12
chr13
chr14
chr15
chr16
chr17
chr18
chr19
chr20
chr21
MDM4
MYCN
GLI2
FGFR3/TACC3
PDGFRA
TERT
MYB
EGFR
CDK6
MET
KIAA1549/BRAF
FGFR1/TACC1
MYBL1
MYC
CDKN2A/B
PTCH1
PTEN
MGMT
CCND1
CCND2
CDK4
MDM2
RB1
TP53
NF1
PPM1D
C19MC
SMARCB1

#4

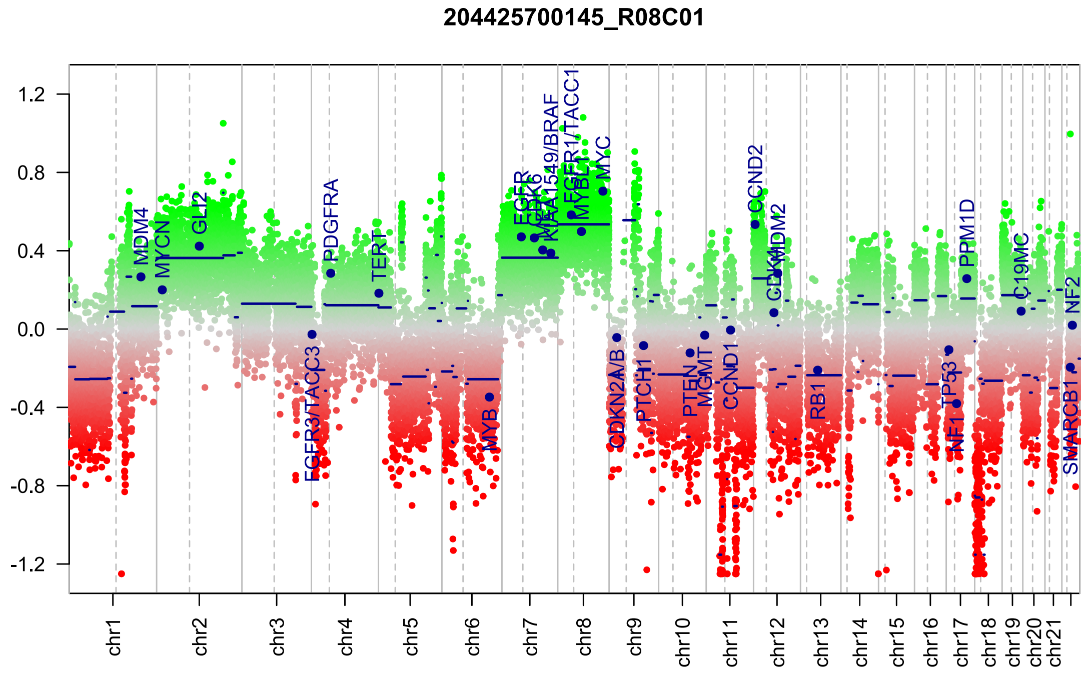
204425700145_R08C01
1.2
0.8
0.4
0.0
-0.4
-0.8
-1.2
chr1
chr2
chr3
chr4
chr5
chr6
chr7
chr8
chr9
chr10
chr11
chr12
chr13
chr14
chr15
chr16
chr17
chr18
chr19
chr20
chr21
MDM4
MYCN
GLI2
FGFR3/TACC3
PDGFRA
TERT
MYB
EGFR
KIAA1549/BRAF
FGFR1/TACC1
MYC
CDKN2A/B
PTCH1
PTEN
MGMT
CCND1
CCND2
MDM2
RB1
TP53
NF1
PPM1D
C19MC
NF2
SMARCB1

#5

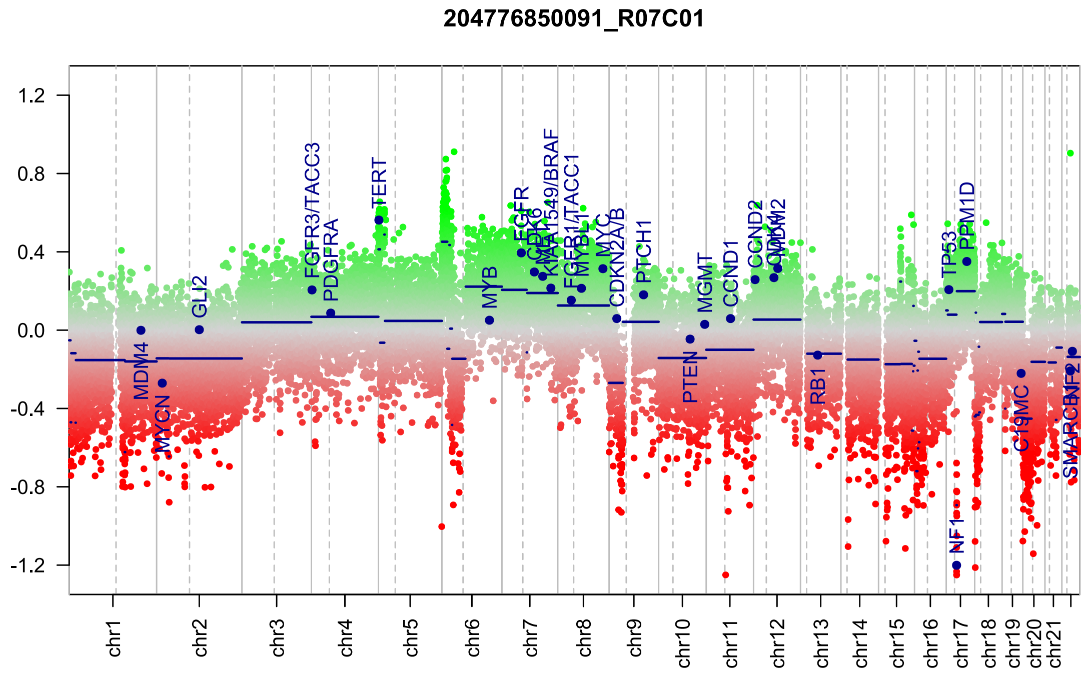
204776850091_R07C01
1.2
0.8
0.4
0.0
-0.4
-0.8
-1.2
chr1
chr2
chr3
chr4
chr5
chr6
chr7
chr8
chr9
chr10
chr11
chr12
chr13
chr14
chr15
chr16
chr17
chr18
chr19
chr20
chr21
MDM4
MYCN
GLI2
FGFR3/TACC3
PDGFRA
TERT
MYB
EGFR
KIAA1549/BRAF
FGFR1/TACC1
MYC
CDKN2A/B
PTCH1
PTEN
MGMT
CCND1
CCND2
MDM2
RB1
TP53
NF1
PPM1D
C19MC

#6

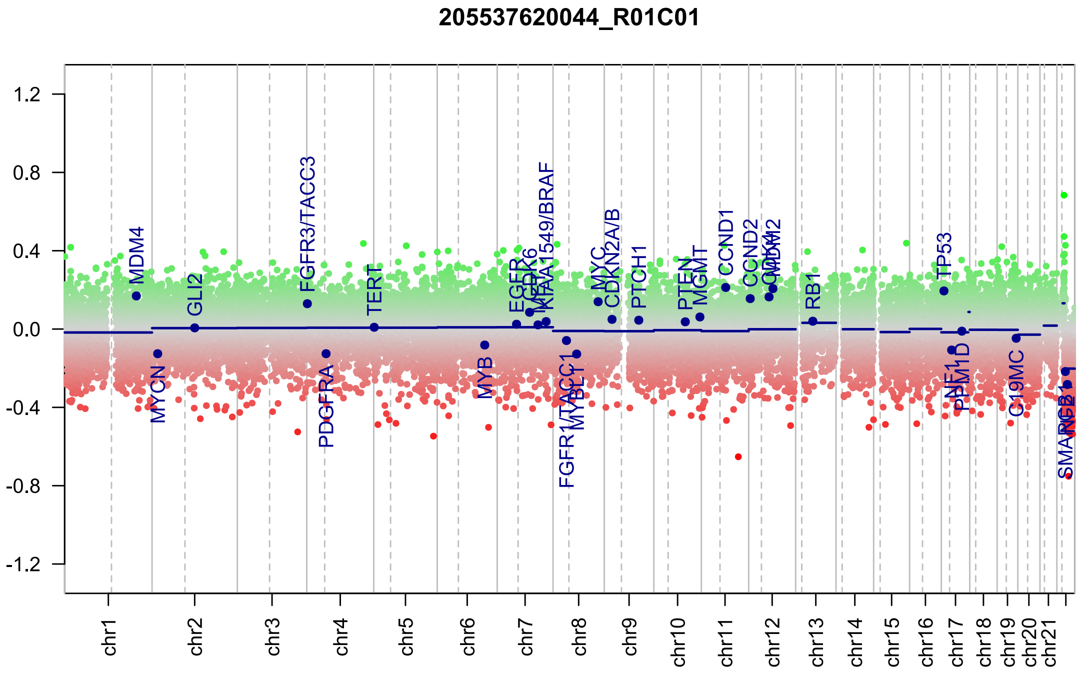
205537620044_R01C01
1.2
0.8
0.4
0.0
-0.4
-0.8
-1.2
chr1
chr2
chr3
chr4
chr5
chr6
chr7
chr8
chr9
chr10
chr11
chr12
chr13
chr14
chr15
chr16
chr17
chr18
chr19
chr20
chr21
MDM4
MYCN
GLI2
FGFR3/TACC3
PDGFRA
TERT
MYB
EGFR
KIAA1549/BRAF
MYC
CDKN2A/B
PTCH1
PTEN
MGMT
CCND1
CCND2
RB1
TP53
C19MC

#7

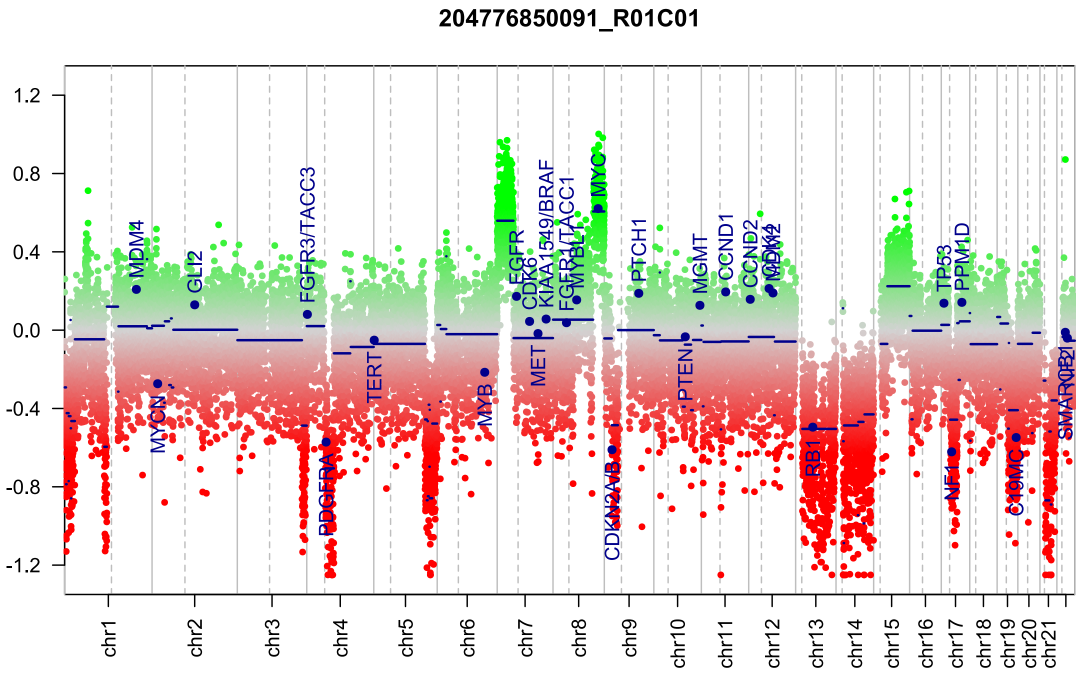
204776850091_R01C01
1.2
0.8
0.4
0.0
-0.4
-0.8
-1.2
chr1
chr2
chr3
chr4
chr5
chr6
chr7
chr8
chr9
chr10
chr11
chr12
chr13
chr14
chr15
chr16
chr17
chr18
chr19
chr20
chr21
MDM4
MYCN
GLI2
FGFR3/TACC3
PDGFRA
TERT
MYB
EGFR
CDK6
MET
KIAA1549/BRAF
FGFR1/TACC1
MYC
CDKN2A/B
PTCH1
PTEN
MGMT
CCND1
CCND2
RB1
TP53
PPM1D
NF1
C19MC

#8

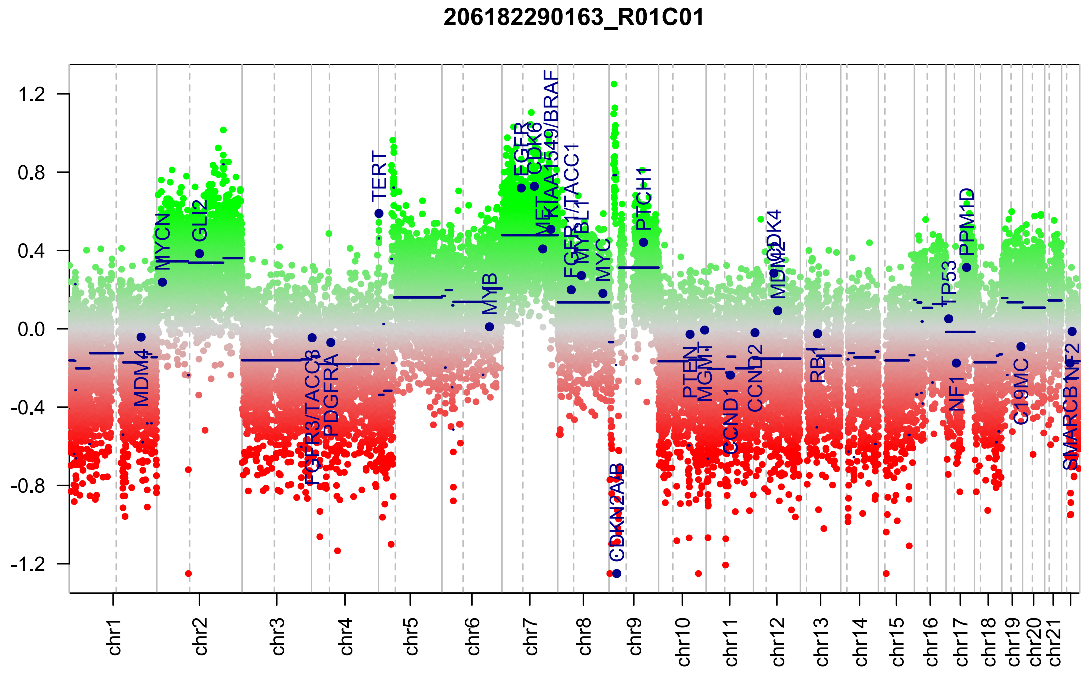
206182290163_R01C01
1.2
0.8
0.4
0.0
-0.4
-0.8
-1.2
chr1
chr2
chr3
chr4
chr5
chr6
chr7
chr8
chr9
chr10
chr11
chr12
chr13
chr14
chr15
chr16
chr17
chr18
chr19
chr20
chr21
MYCN
GLI2
MDM4
TERT
EGFR
CDK6
KIAA1549/BRAF
FGFR1/TACC1
MET
MYB
MYC
PTCH1
CDKN2A/B
PTEN
MGMT
CCND1
CCND2
CDK4
MDM2
RB1
TP53
PPM1D
NF1
C19MC
NF2
SMARCB1
FGFR3/TACC3
PDGFRA

#9

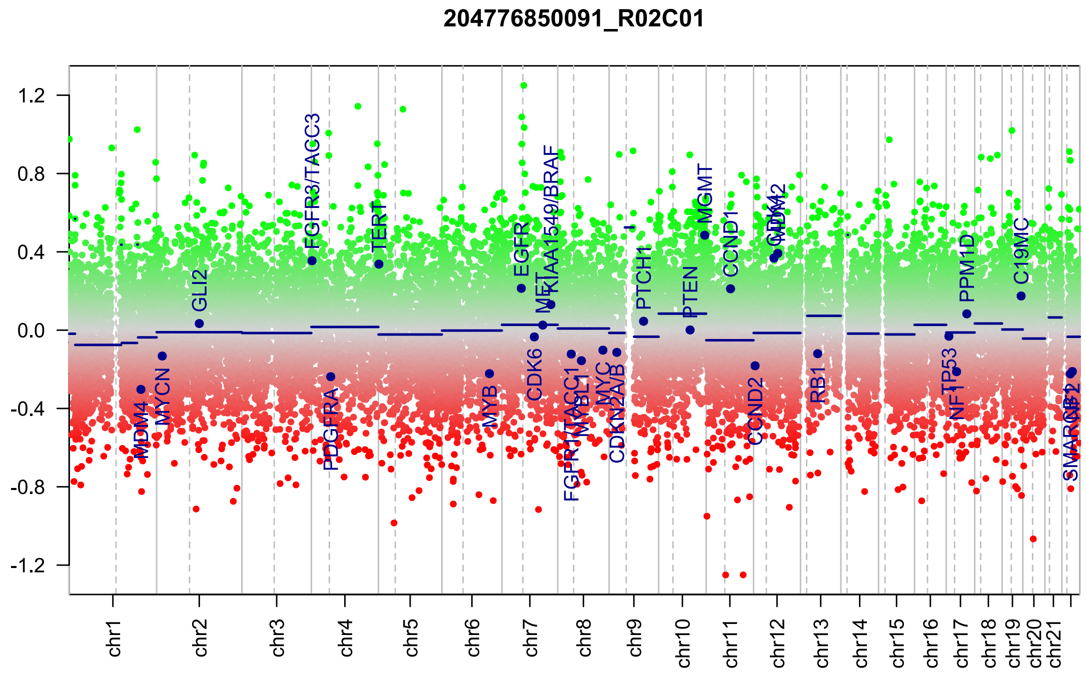
204776850091_R02C01
1.2
0.8
0.4
0.0
-0.4
-0.8
-1.2
chr1
chr2
chr3
chr4
chr5
chr6
chr7
chr8
chr9
chr10
chr11
chr12
chr13
chr14
chr15
chr16
chr17
chr18
chr19
chr20
chr21
FGFR3/TACC3
TERT
EGFR
KIAA1549/BRAF
MET
GLI2
MDM4
MYCN
PDGFRA
MYB
CDK6
FGFR1/TACC1
MYC
CDKN2A/B
PTCH1
PTEN
MGMT
CCND1
CDK4
MDM2
CCND2
RB1
TP53
NF1
PPM1D
C19MC
SMARCB1
NF2

#10

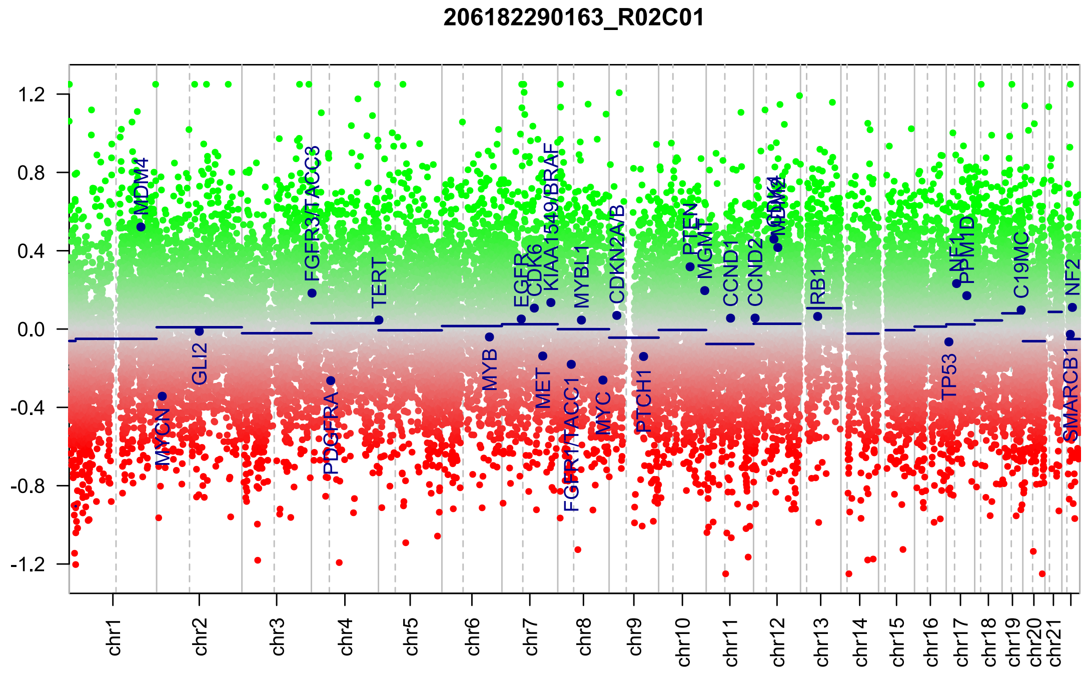
206182290163_R02C01
1.2
0.8
0.4
0.0
-0.4
-0.8
-1.2
MDM4
FGFR3/TACC3
TERT
EGFR
CDK6
KIAA1549/BRAF
MYBL1
CDKN2A/B
PTEN
MGMT
CCND1
CCND2
RB1
NF1
PPM1D
C19MC
NF2
GLI2
MYCN
PDGFRA
MYB
MET
FGFR1/TACC1
MYC
PTCH1
TP53
SMARCB1
chr1
chr2
chr3
chr4
chr5
chr6
chr7
chr8
chr9
chr10
chr11
chr12
chr13
chr14
chr15
chr16
chr17
chr18
chr19
chr20
chr21

#11

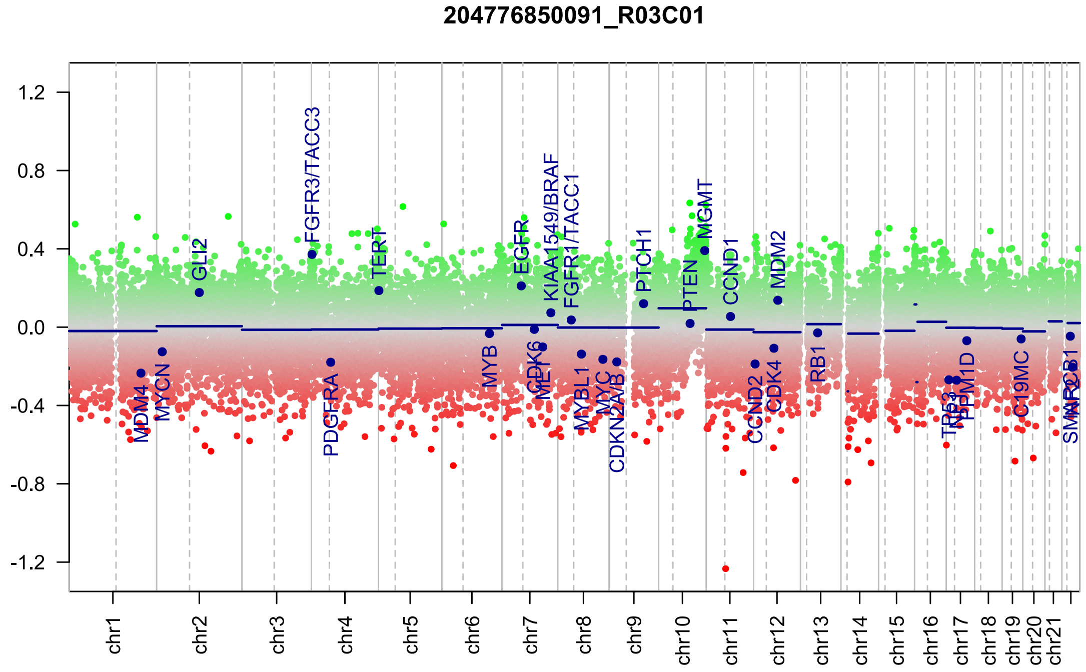
204776850091_R03C01
1.2
0.8
0.4
0.0
-0.4
-0.8
-1.2
GLI2
FGFR3/TACC3
TERT
EGFR
KIAA1549/BRAF
FGFR1/TACC1
PTCH1
PTEN
MGMT
CCND1
MDM2
MDM4
MYCN
PDGFRA
MYB
CDK6
MET
MYBL1
MYC
CDKN2A/B
CCND2
CDK4
RB1
TP53
PPM1D
C19MC
SMARCB1
chr1
chr2
chr3
chr4
chr5
chr6
chr7
chr8
chr9
chr10
chr11
chr12
chr13
chr14
chr15
chr16
chr17
chr18
chr19
chr20
chr21

#12

204776850091_R04C01

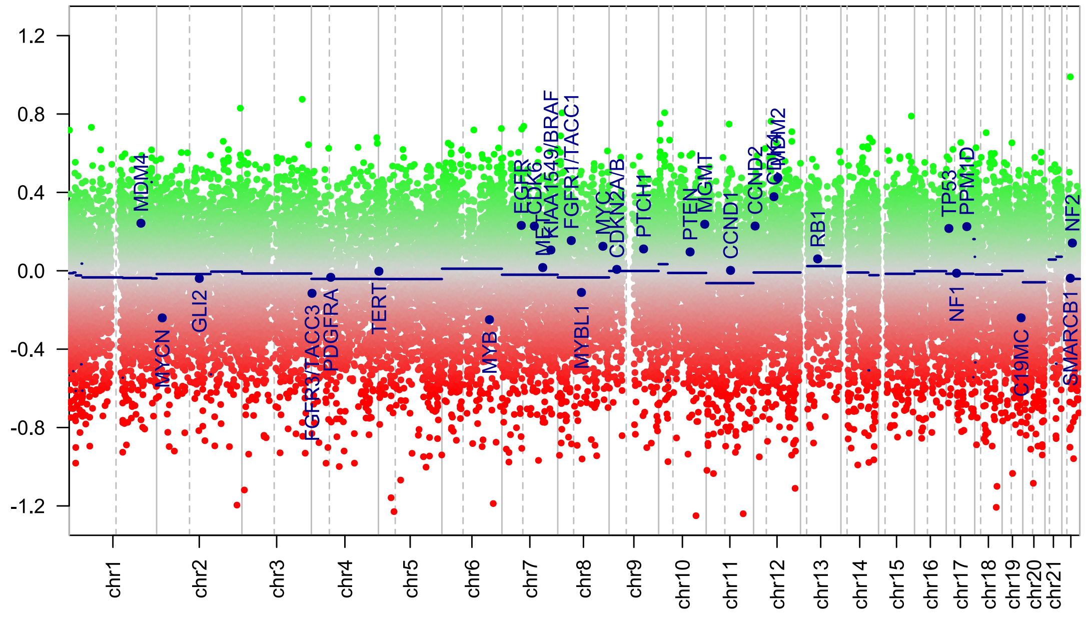

#13

204776850091_R05C01

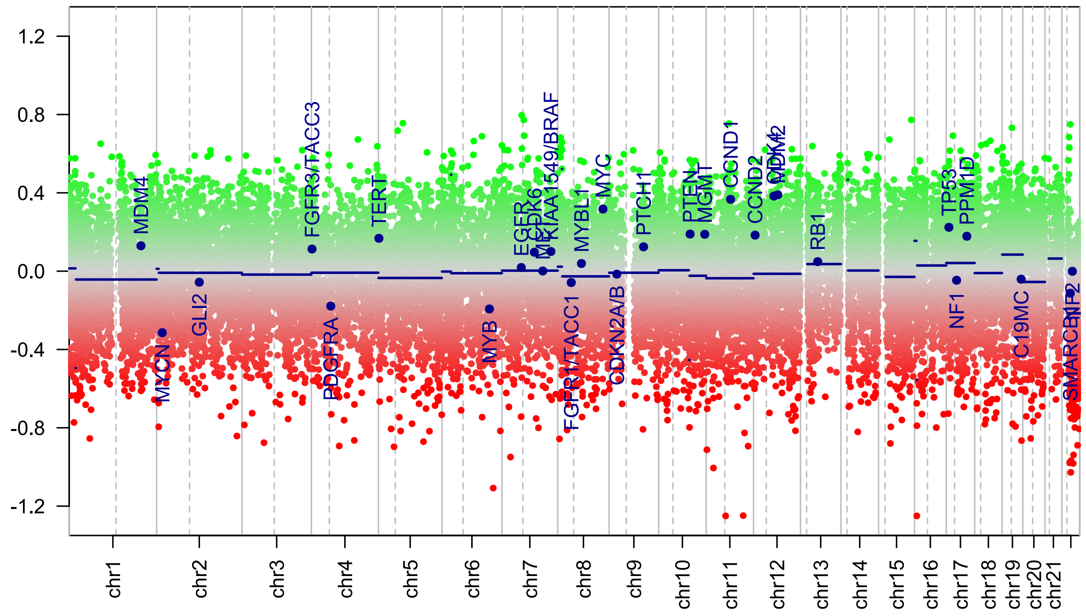

#14

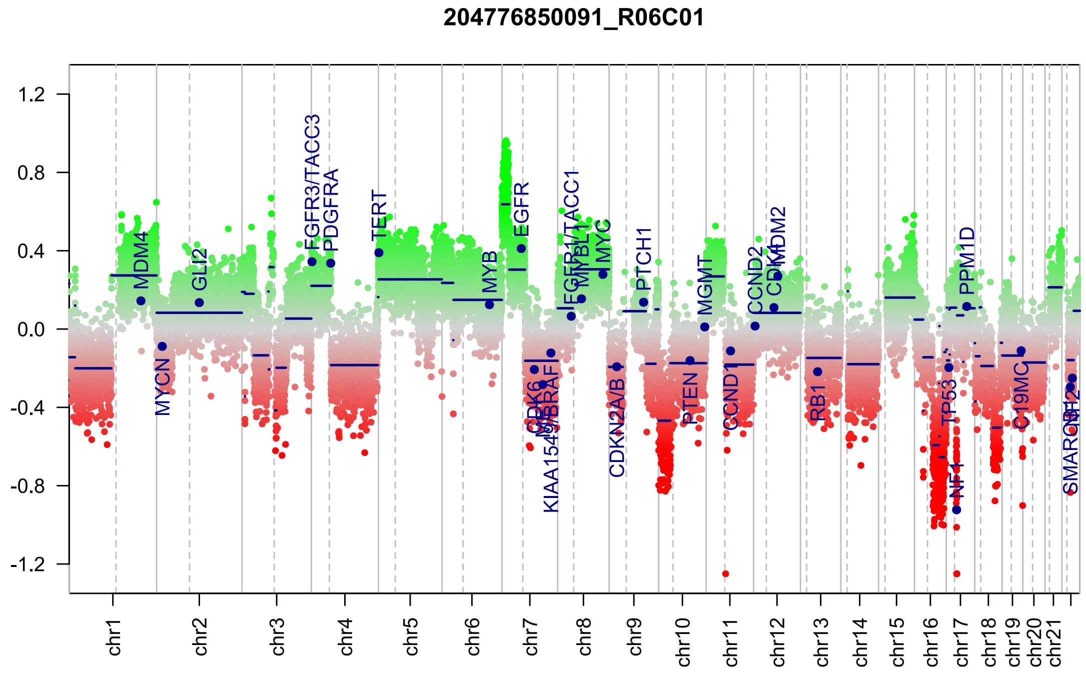
204776850091_R06C01
1.2
0.8
0.4
0.0
-0.4
-0.8
-1.2
chr1
chr2
chr3
chr4
chr5
chr6
chr7
chr8
chr9
chr10
chr11
chr12
chr13
chr14
chr15
chr16
chr17
chr18
chr19
chr20
chr21

#15

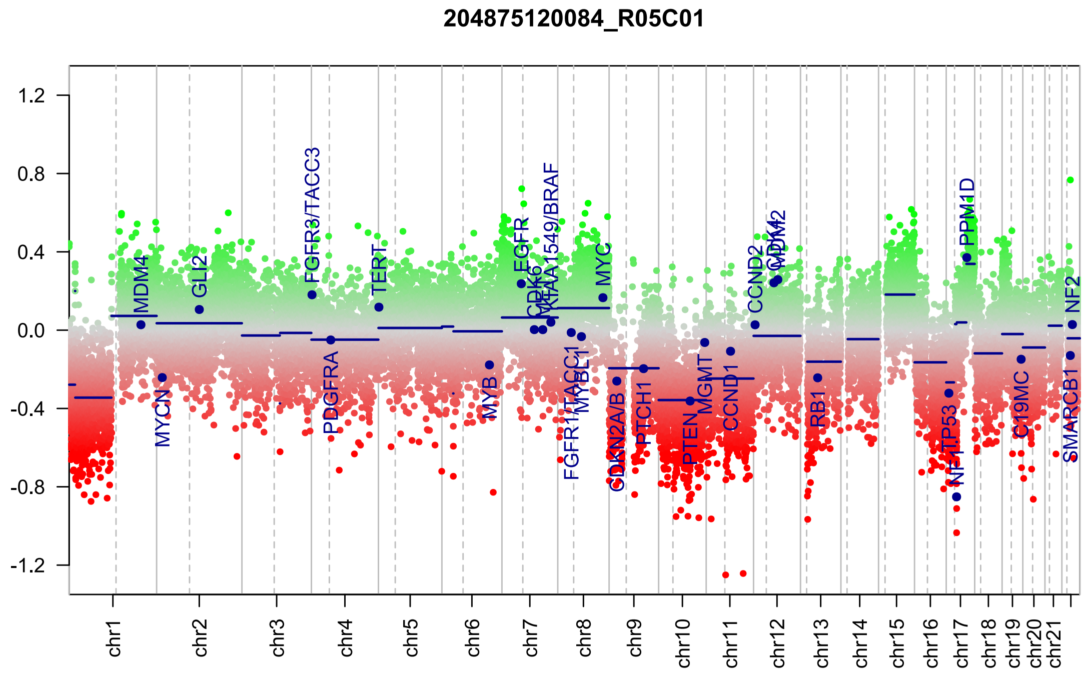
204875120084_R05C01
1.2
0.8
0.4
0.0
-0.4
-0.8
-1.2
chr1
chr2
chr3
chr4
chr5
chr6
chr7
chr8
chr9
chr10
chr11
chr12
chr13
chr14
chr15
chr16
chr17
chr18
chr19
chr20
chr21

#16

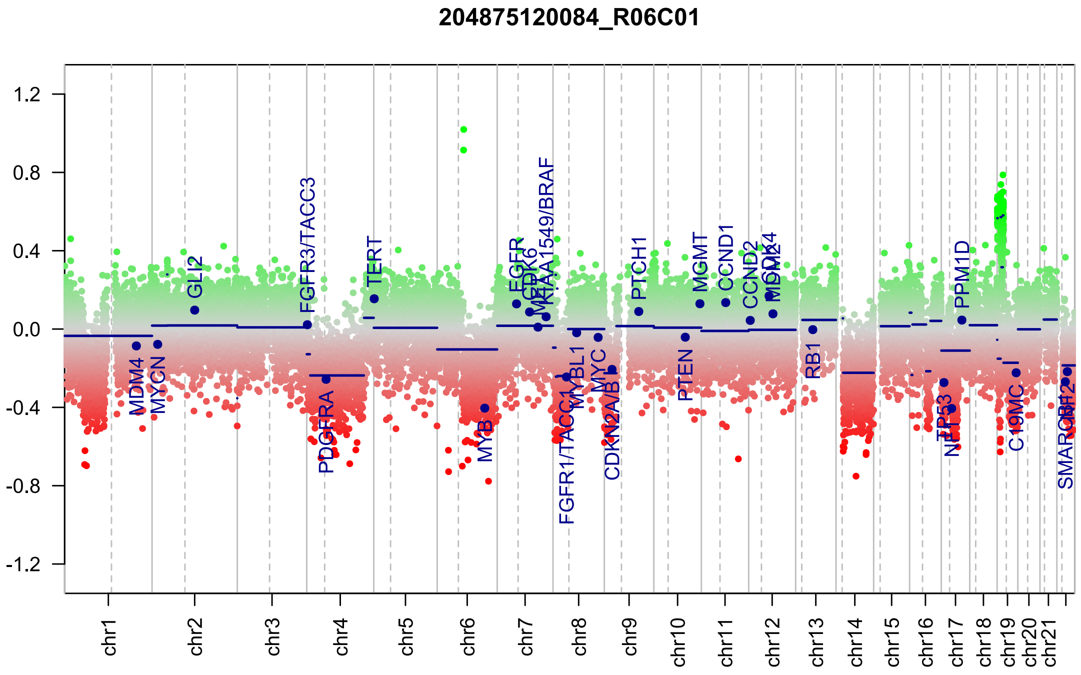
204875120084_R06C01
1.2
0.8
0.4
0.0
-0.4
-0.8
-1.2
chr1
chr2
chr3
chr4
chr5
chr6
chr7
chr8
chr9
chr10
chr11
chr12
chr13
chr14
chr15
chr16
chr17
chr18
chr19
chr20
chr21
GLI2
FGFR3/TACC3
TERT
EGFR
KIAA1549/BRAF
PTCH1
MGMT
CCND1
CCND2
PPM1D
MDM4
MYCN
PDGFRA
MYB
PTEN
RB1
TP53
C19MC

#17

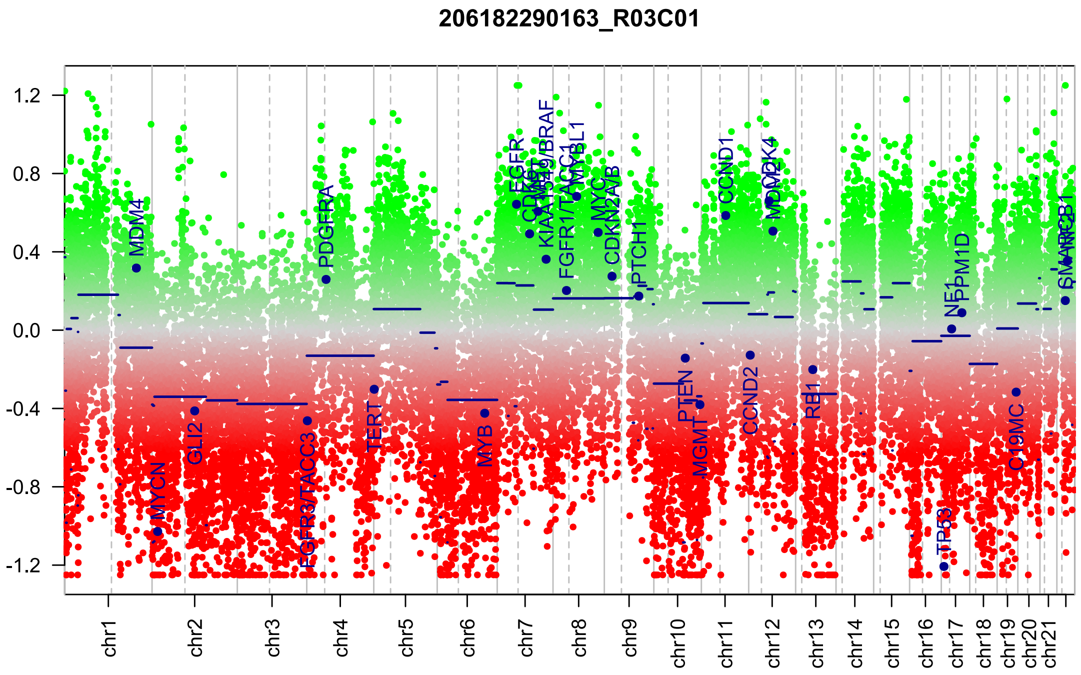
206182290163_R03C01
1.2
0.8
0.4
0.0
-0.4
-0.8
-1.2
chr1
chr2
chr3
chr4
chr5
chr6
chr7
chr8
chr9
chr10
chr11
chr12
chr13
chr14
chr15
chr16
chr17
chr18
chr19
chr20
chr21
MDM4
PDGFRA
EGFR
KIAA1549/BRAF
CCND1
PTCH1
PPM1D
MYCN
GLI2
FGFR3/TACC3
TERT
MYB
PTEN
MGMT
CCND2
C19MC

#18

205537620044_R02C01

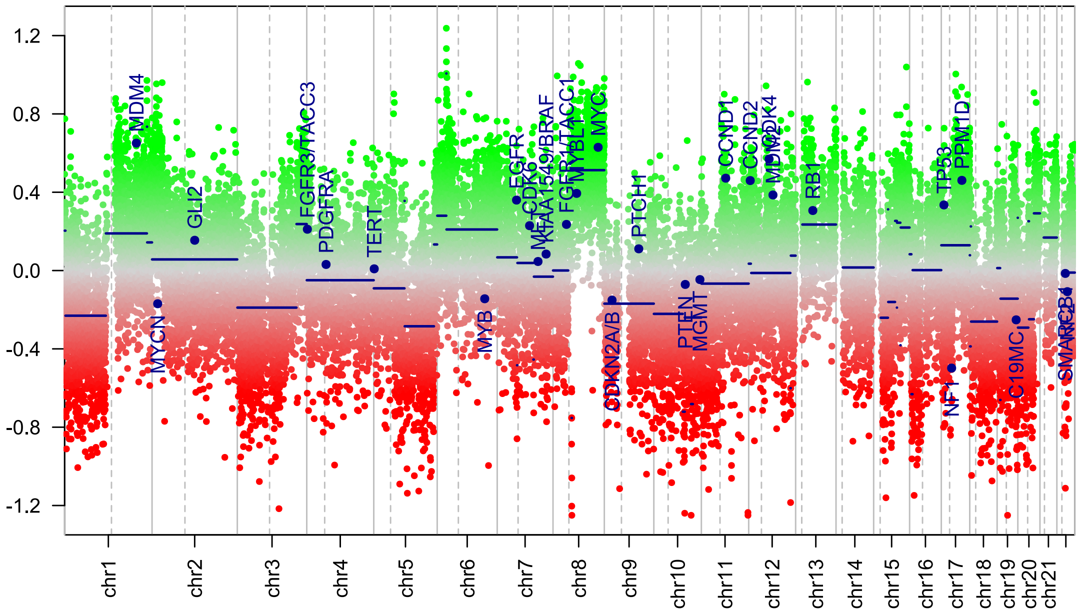

#19

204792780085_R06C01

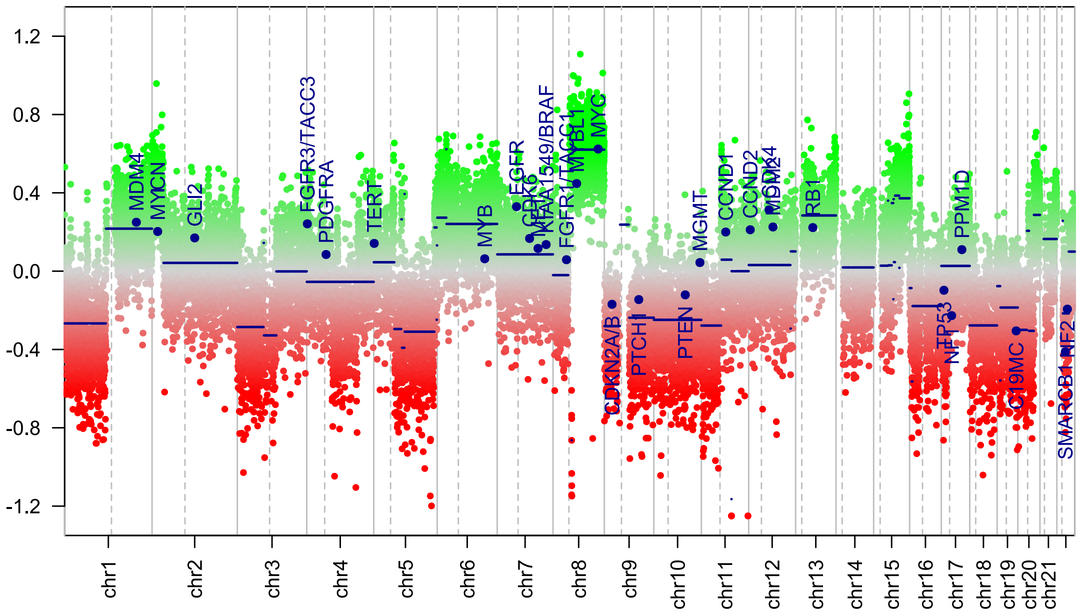

#20

204792780085_R07C01

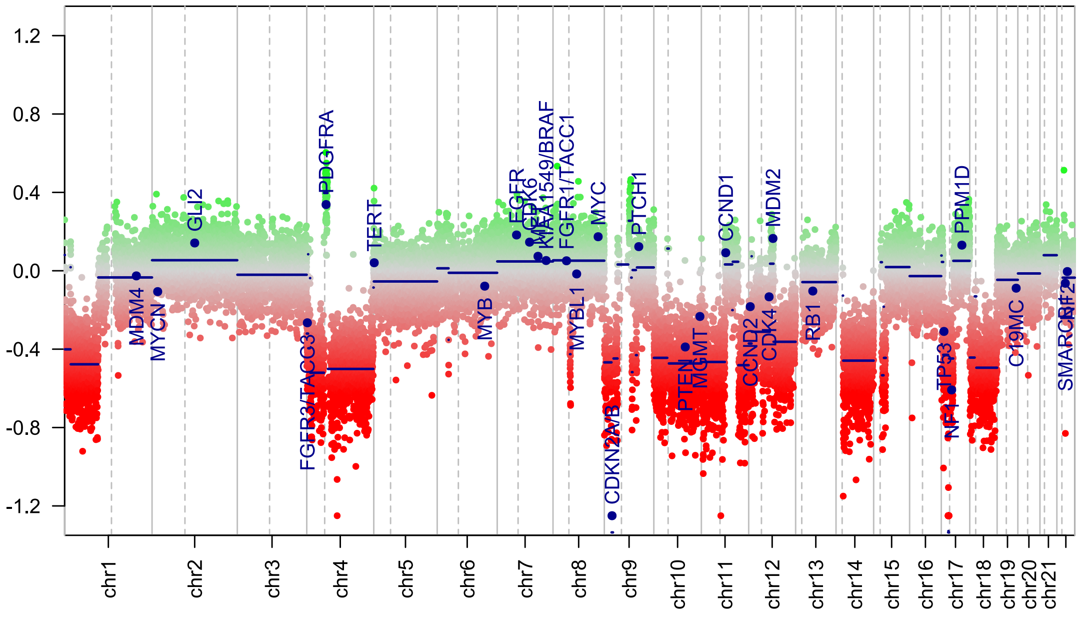

#21

204792780085_R08C01

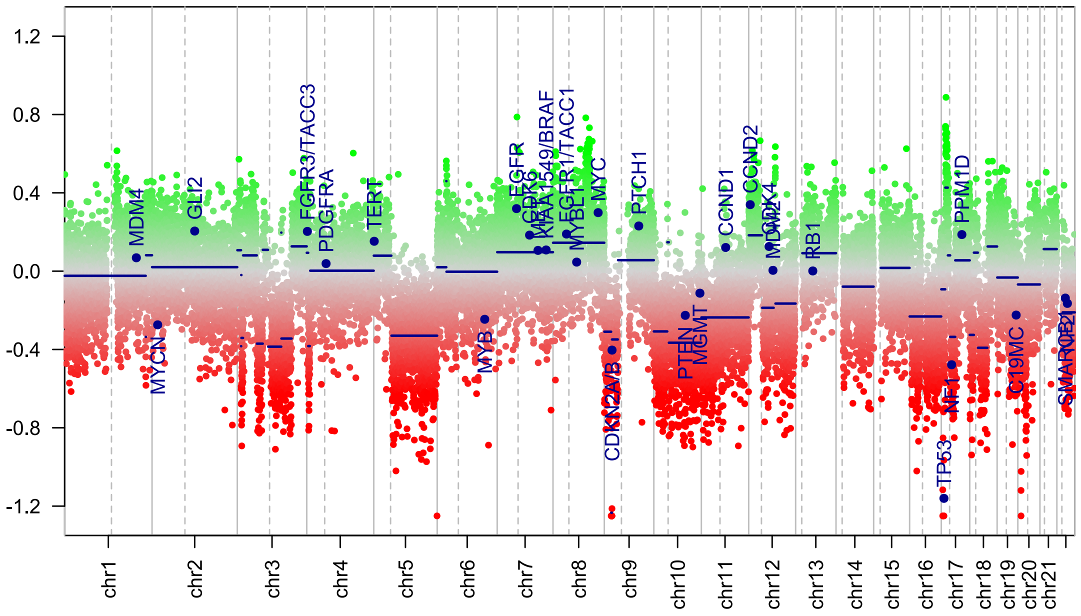

#22

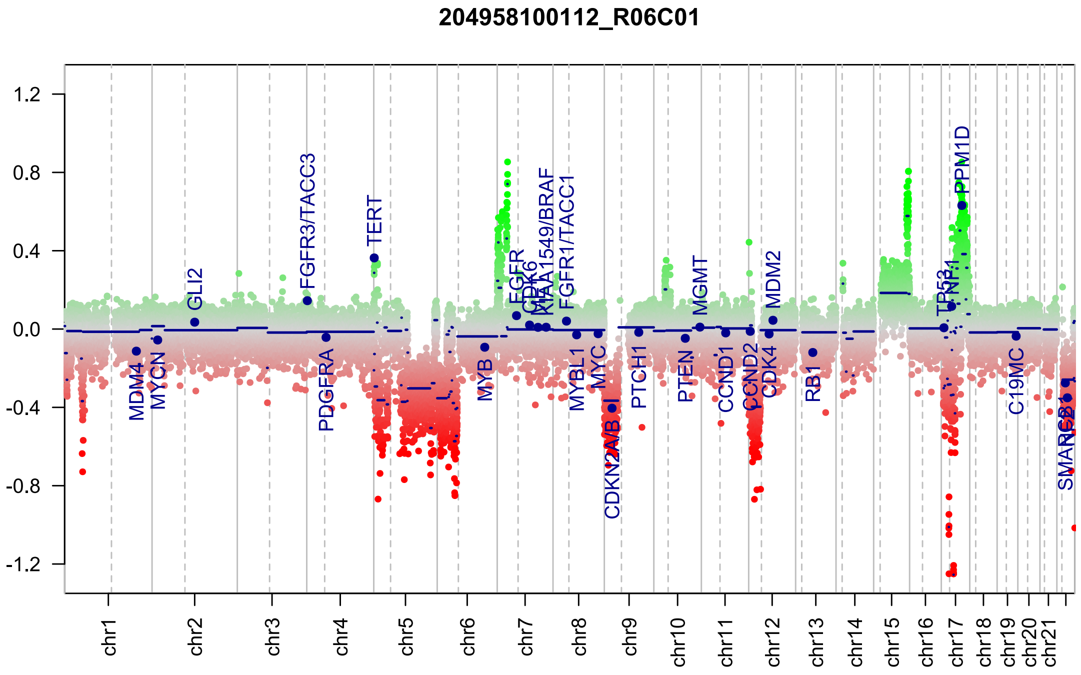
204958100112_R06C01
1.2
0.8
0.4
0.0
-0.4
-0.8
-1.2
chr1 chr2 chr3 chr4 chr5 chr6 chr7 chr8 chr9 chr10 chr11 chr12 chr13 chr14 chr15 chr16 chr17 chr18 chr19 chr20 chr21

#23

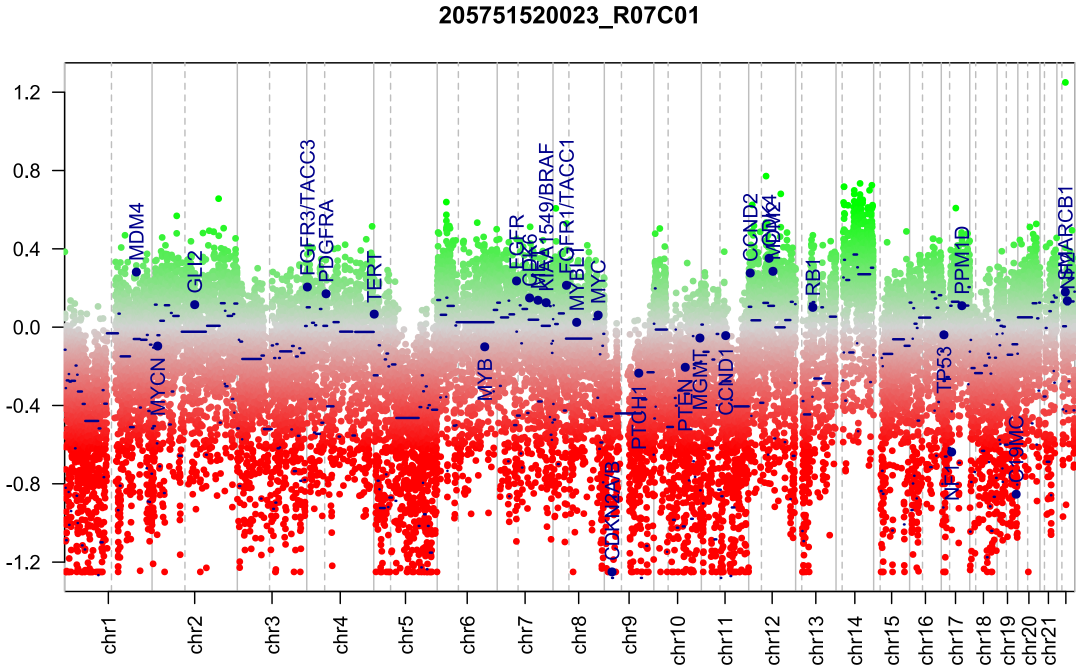
205751520023_R07C01
1.2
0.8
0.4
0.0
-0.4
-0.8
-1.2
chr1 chr2 chr3 chr4 chr5 chr6 chr7 chr8 chr9 chr10 chr11 chr12 chr13 chr14 chr15 chr16 chr17 chr18 chr19 chr20 chr21

#24

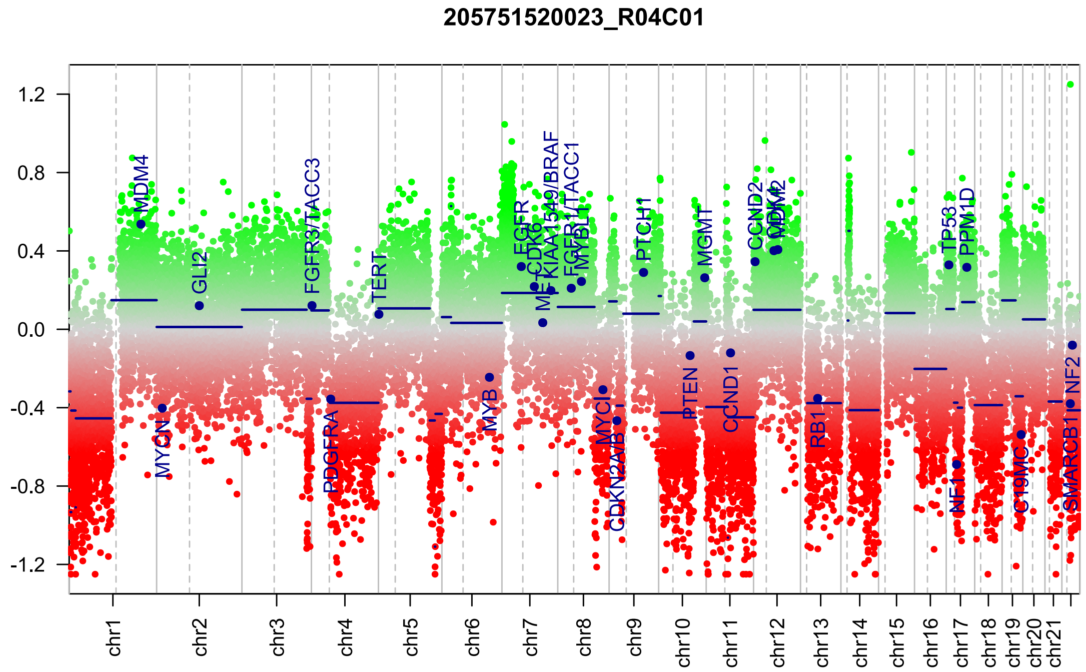
205751520023_R04C01
1.2
0.8
0.4
0.0
-0.4
-0.8
-1.2
MDM4
MYCN
GLI2
FGFR3/TACC3
PDGFRA
TERT
MYB
EGFR
CDK6
MET
KIAA1549/BRAF
FGFR1/TACC1
MYBL1
MYC
CDKN2A/B
PTCH1
PTEN
MGMT
CCND1
CCND2
CDK4
MDM2
RB1
TP53
PPM1D
NF1
C19MC
SMARCB1
NF2
chr1
chr2
chr3
chr4
chr5
chr6
chr7
chr8
chr9
chr10
chr11
chr12
chr13
chr14
chr15
chr16
chr17
chr18
chr19
chr20
chr21

#25

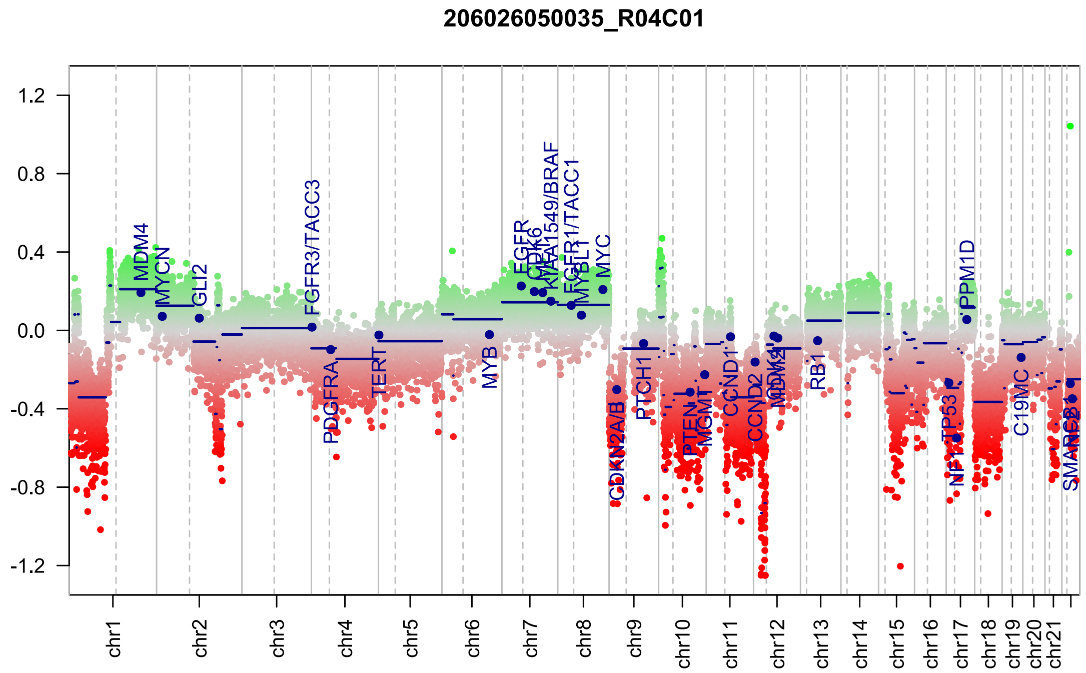
206026050035_R04C01
1.2
0.8
0.4
0.0
-0.4
-0.8
-1.2
MDM4
MYCN
GLI2
FGFR3/TACC3
PDGFRA
TERT
MYB
EGFR
CDK6
KIAA1549/BRAF
FGFR1/TACC1
MYBL1
MYC
CDKN2A/B
PTCH1
PTEN
MGMT
CCND1
CCND2
RB1
TP53
PPM1D
NF1
C19MC
chr1
chr2
chr3
chr4
chr5
chr6
chr7
chr8
chr9
chr10
chr11
chr12
chr13
chr14
chr15
chr16
chr17
chr18
chr19
chr20
chr21

#26

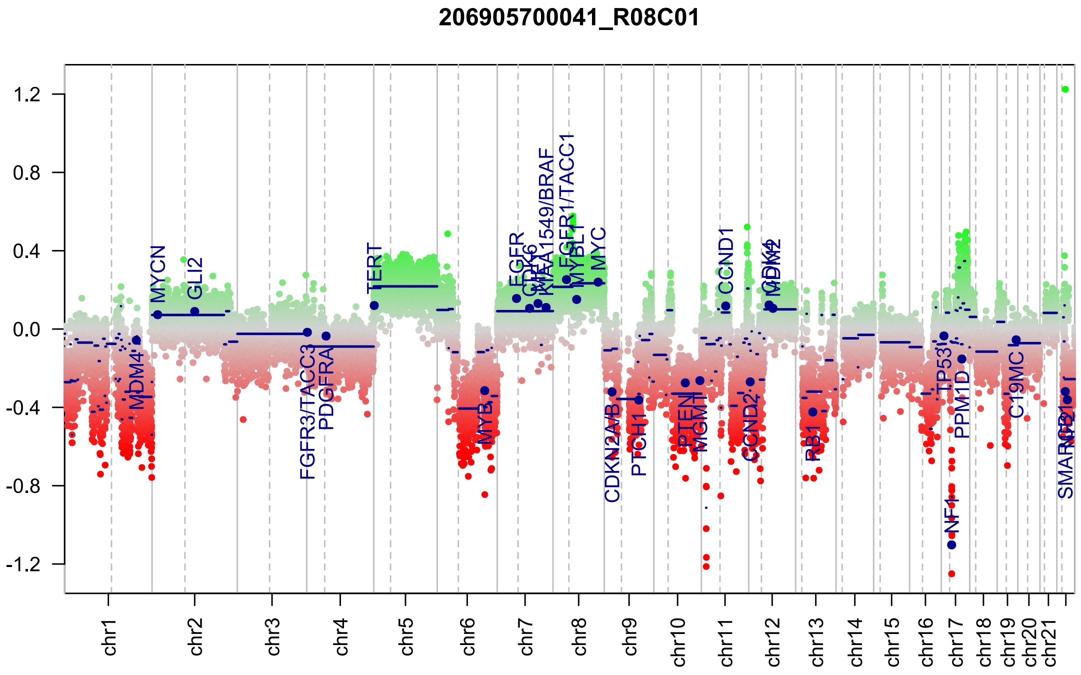
206905700041_R08C01
1.2
0.8
0.4
0.0
-0.4
-0.8
-1.2
MYCN
GLI2
MDM4
FGFR3/TACC3
PDGFRA
TERT
MYB
EGFR
KIAA1549/BRAF
FGFR1/TACC1
MYC
CDKN2A/B
PTCH1
PTEN
CCND1
CCND2
MDM2
RB1
TP53
PPM1D
NF1
C19MC
SMARCB1
NF2
chr1
chr2
chr3
chr4
chr5
chr6
chr7
chr8
chr9
chr10
chr11
chr12
chr13
chr14
chr15
chr16
chr17
chr18
chr19
chr20
chr21

#27

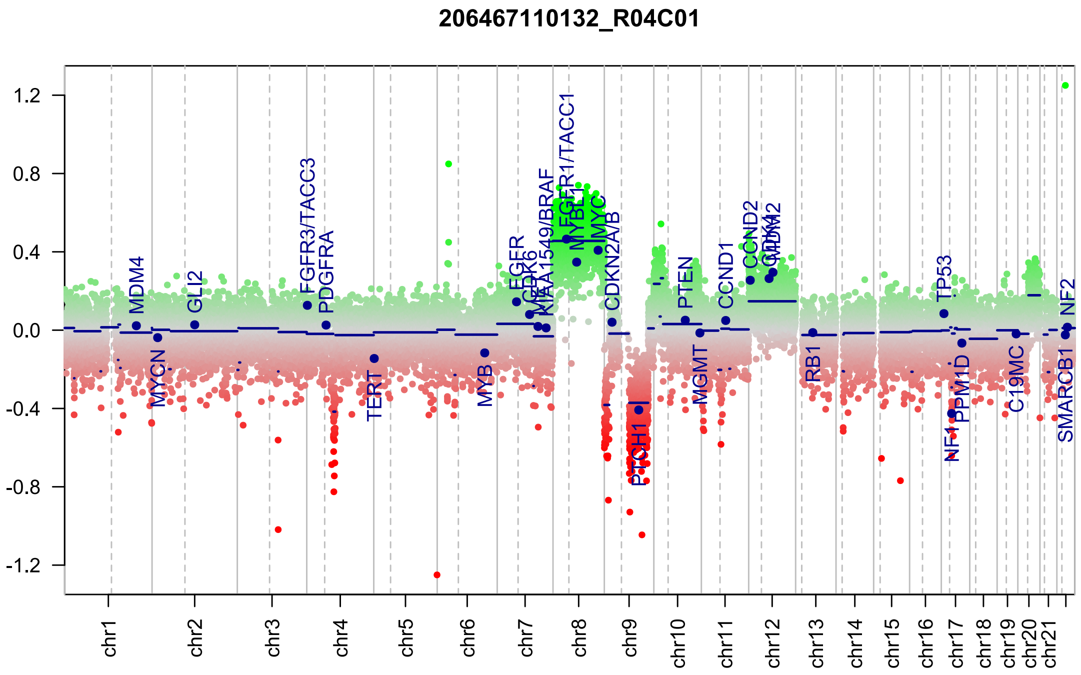
206467110132_R04C01
1.2
0.8
0.4
0.0
-0.4
-0.8
-1.2
MDM4
GLI2
MYCN
FGFR3/TACC3
PDGFRA
TERT
MYB
EGFR
CDK6
KIAA1549/BRAF
FGFR1/TACC1
MYBL1
MYC
CDKN2A/B
PTCH1
PTEN
MGMT
CCND1
CCND2
MDM2
RB1
TP53
PPM1D
NF1
C19MC
NF2
SMARCB1
chr1
chr2
chr3
chr4
chr5
chr6
chr7
chr8
chr9
chr10
chr11
chr12
chr13
chr14
chr15
chr16
chr17
chr18
chr19
chr20
chr21

#28

205751520023_R01C01

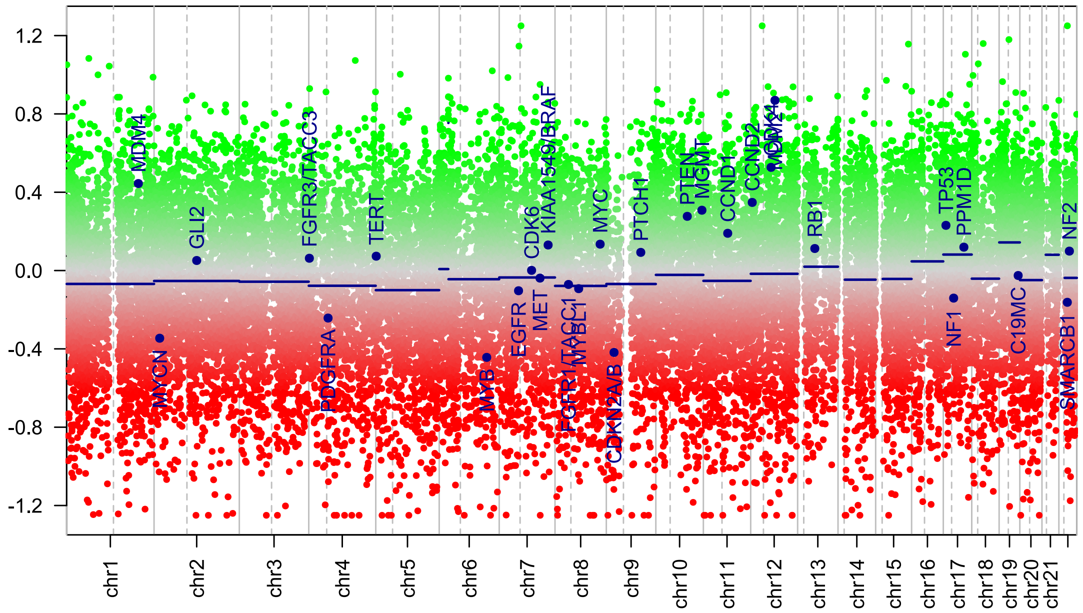

#29

205751520023_R02C01

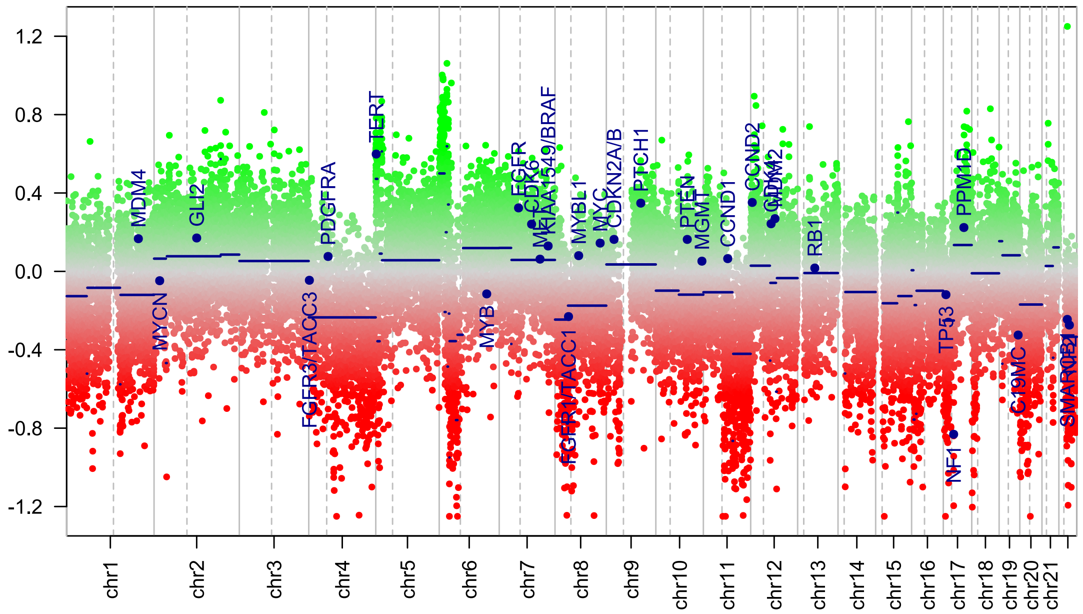

#30

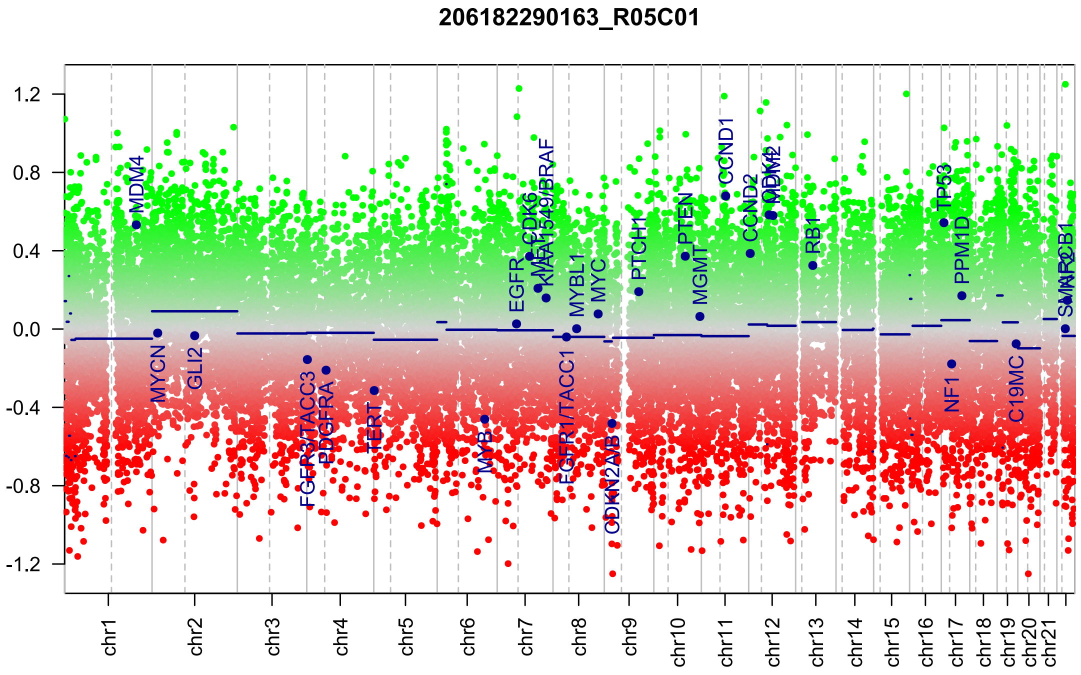
206182290163_R05C01
1.2
0.8
0.4
0.0
-0.4
-0.8
-1.2
chr1
chr2
chr3
chr4
chr5
chr6
chr7
chr8
chr9
chr10
chr11
chr12
chr13
chr14
chr15
chr16
chr17
chr18
chr19
chr20
chr21
MDM4
MYCN
GLI2
FGFR3/TACC3
PDGFRA
TERT
MYB
EGFR
CDK6
KIAA1549/BRAF
FGFR1/TACC1
MYBL1
MYC
CDKN2A/B
PTCH1
PTEN
MGMT
CCND1
CCND2
RB1
TP53
NF1
PPM1D
C19MC

#31

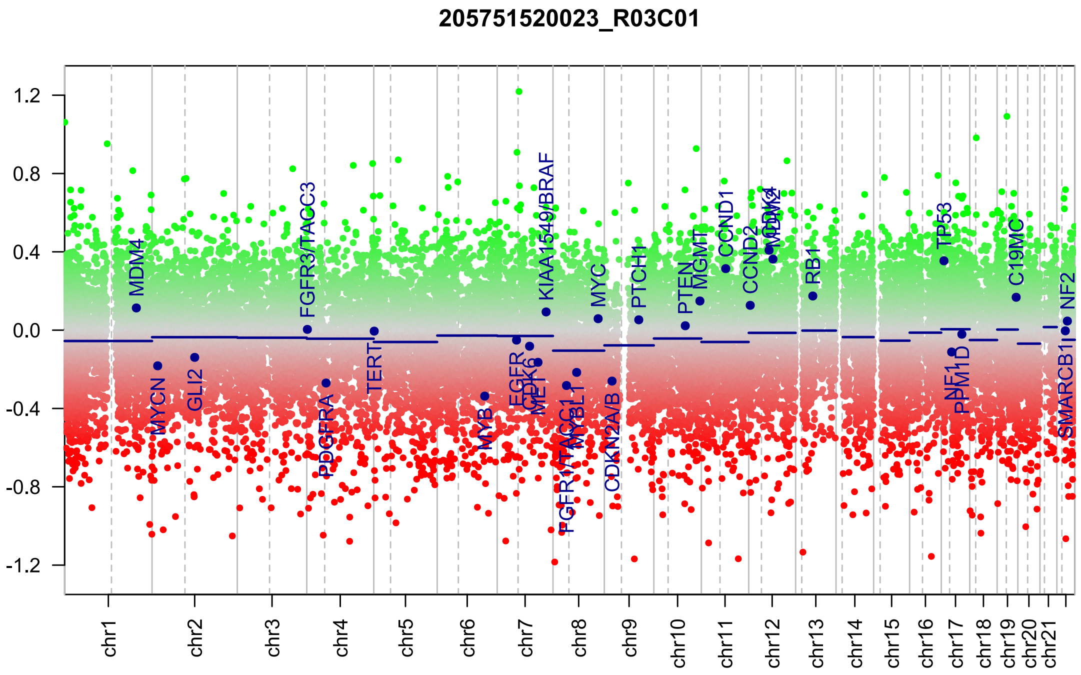
205751520023_R03C01
1.2
0.8
0.4
0.0
-0.4
-0.8
-1.2
chr1
chr2
chr3
chr4
chr5
chr6
chr7
chr8
chr9
chr10
chr11
chr12
chr13
chr14
chr15
chr16
chr17
chr18
chr19
chr20
chr21
MDM4
MYCN
GLI2
FGFR3/TACC3
PDGFRA
TERT
MYB
EGFR
CDK6
MET
KIAA1549/BRAF
FGFR1/TACC1
MYBL1
MYC
CDKN2A/B
PTCH1
PTEN
MGMT
CCND1
CCND2
RB1
TP53
NF1
PPM1D
C19MC
NF2
SMARCB1

#32

205767050149_R02C01

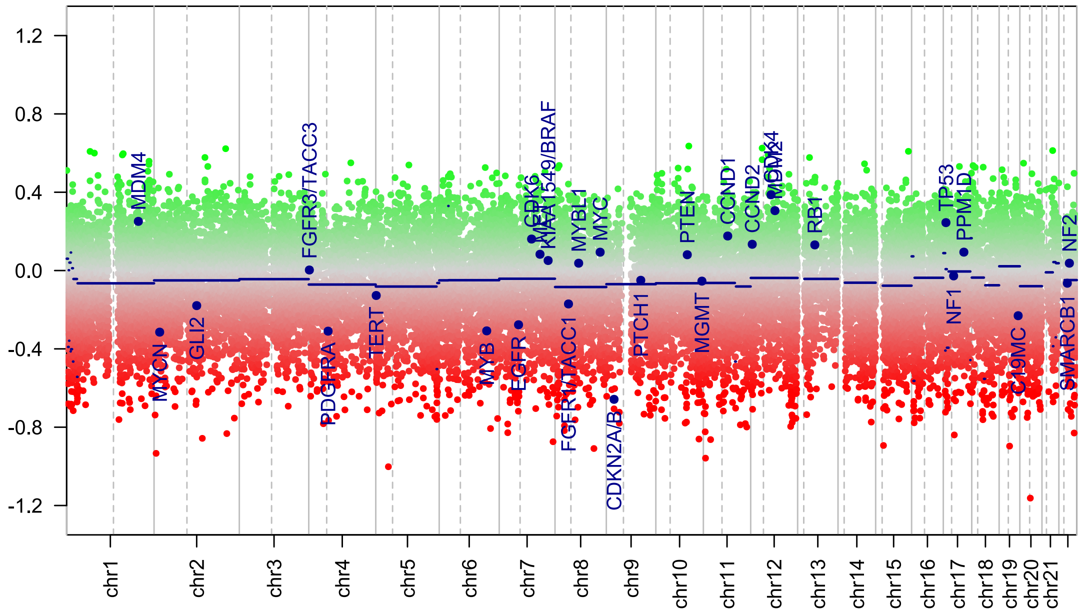

#33

203096660093_R01C01

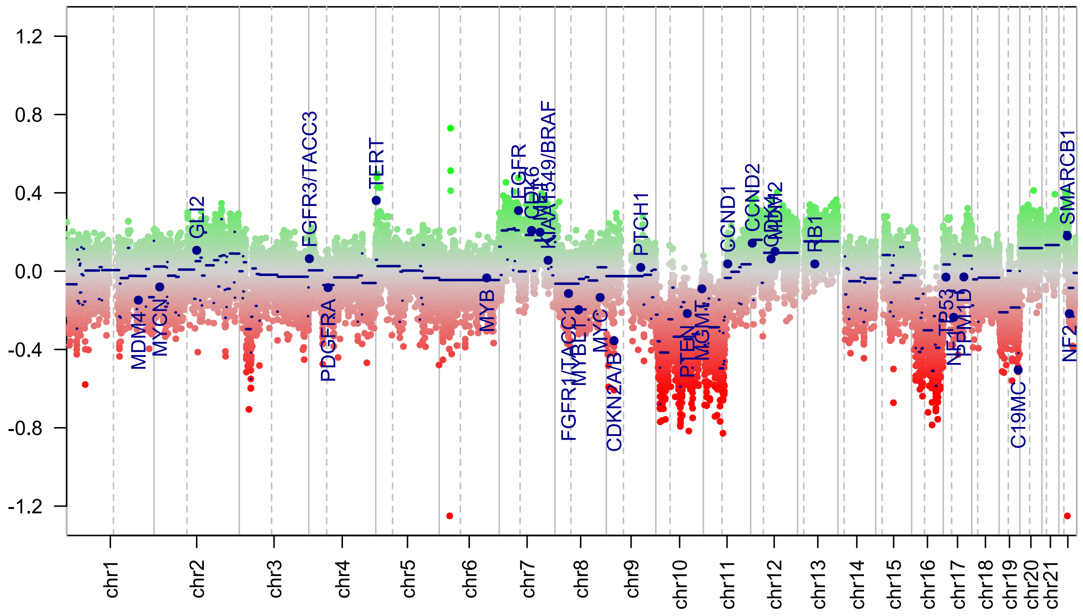

#34

203939360069_R05C01

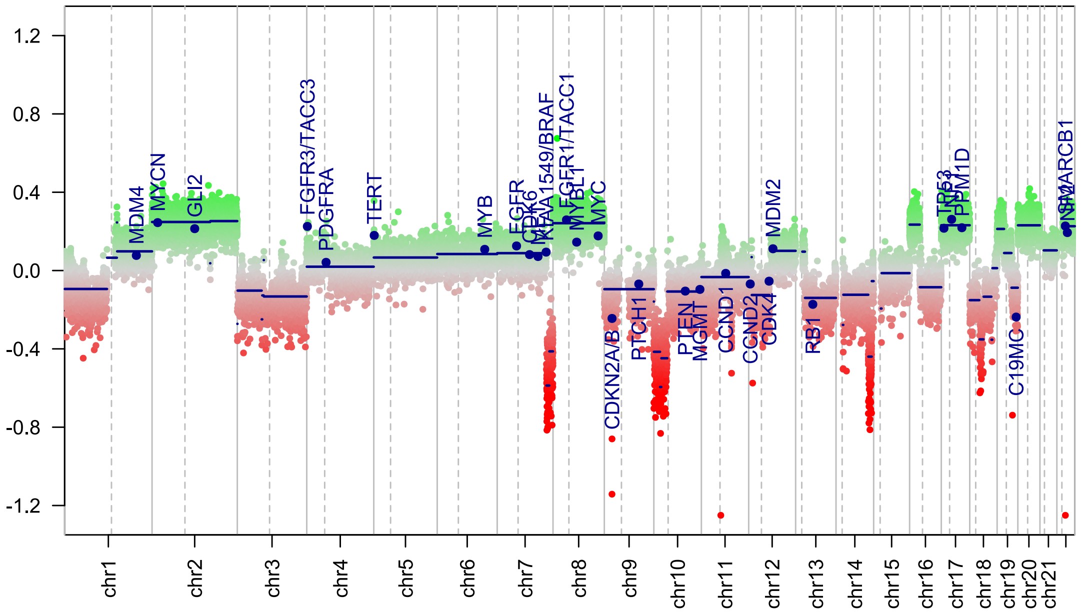

#35

203939360062_R06C01

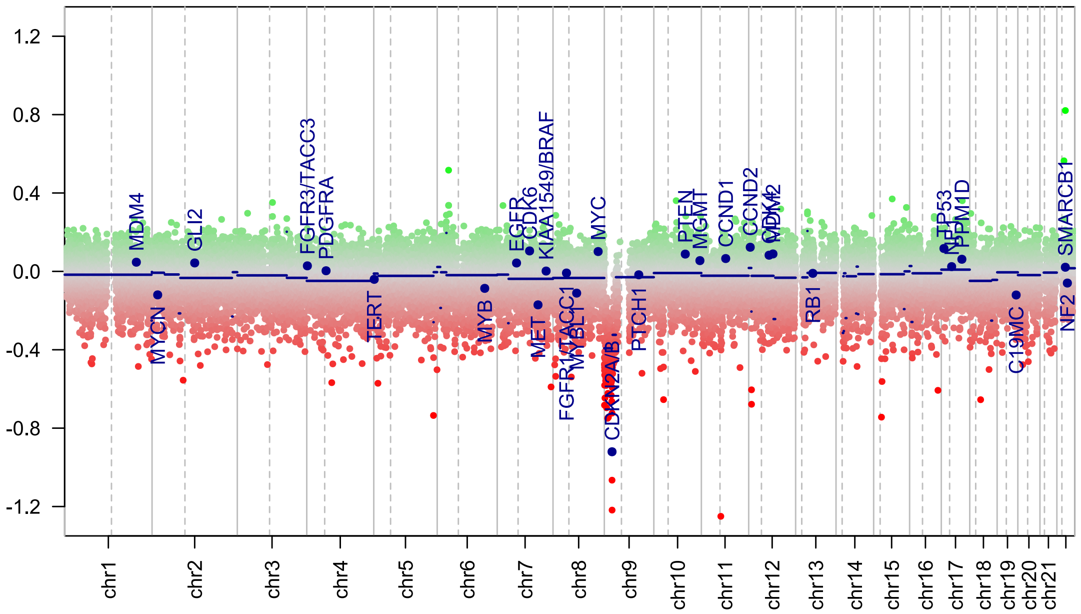

#36

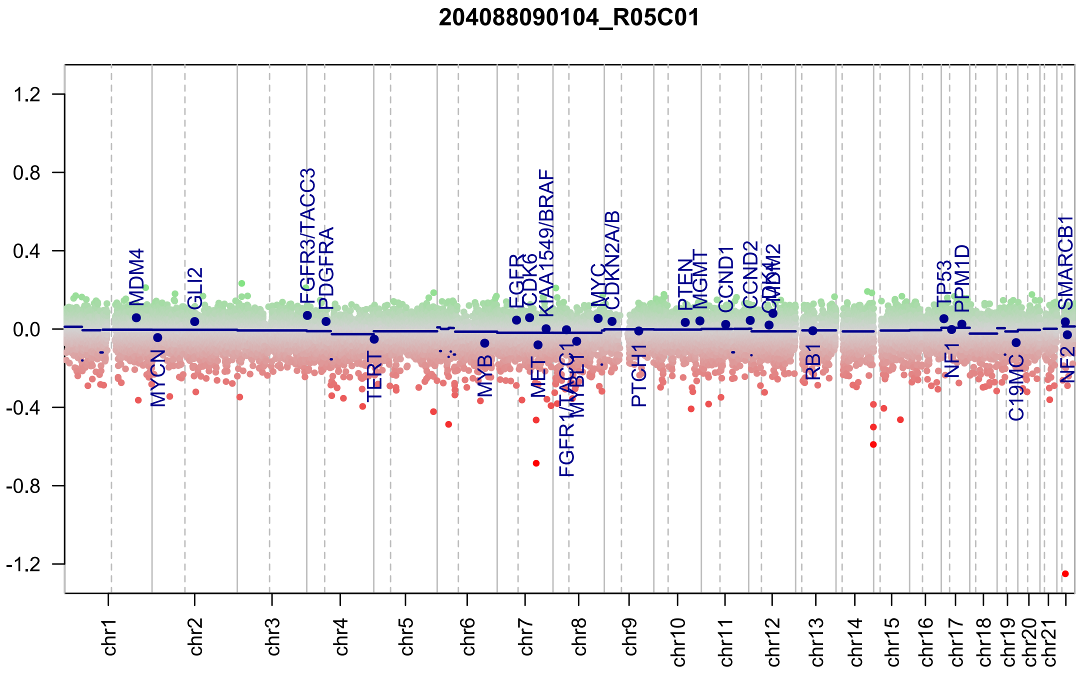

204088090104_R05C01
1.2
0.8
0.4
0.0
-0.4
-0.8
-1.2
MDM4
MYCN
GLI2
FGFR3/TACC3
PDGFRA
TERT
MYB
EGFR
CDK6
KIAA1549/BRAF
MET
FGFR1/TACC1
MYBL1
MYC
CDKN2A/B
PTCH1
PTEN
MGMT
CCND1
CCND2
CDK4
MDM2
RB1
TP53
NF1
PPM1D
C19MC
SMARCB1
NF2
chr1
chr2
chr3
chr4
chr5
chr6
chr7
chr8
chr9
chr10
chr11
chr12
chr13
chr14
chr15
chr16
chr17
chr18
chr19
chr20
chr21

#37

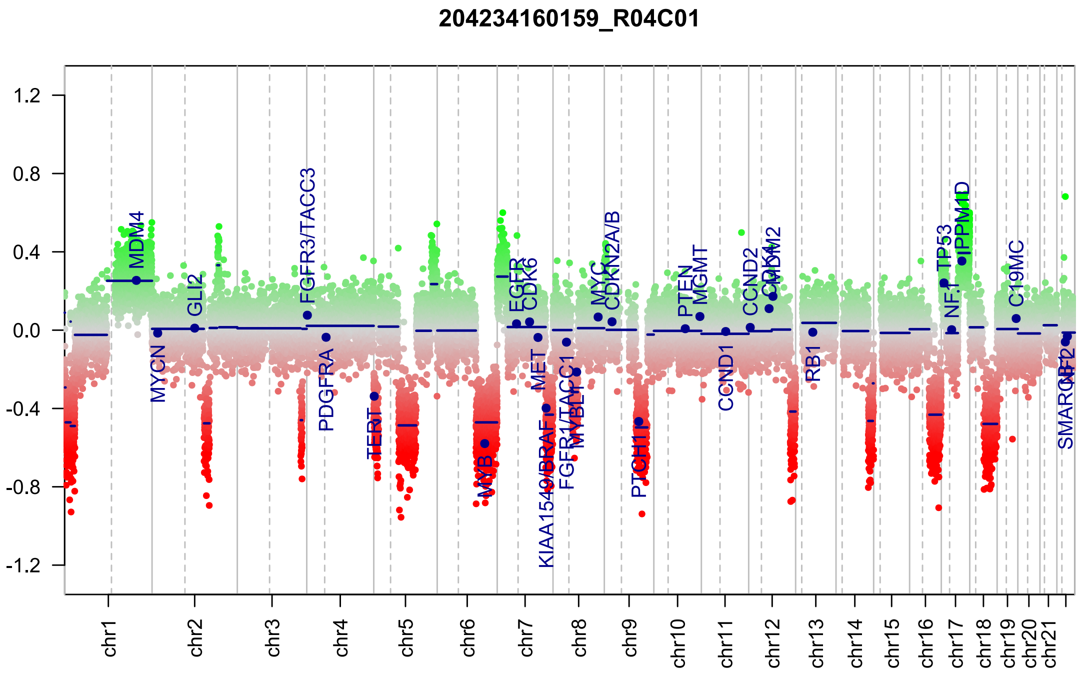

204234160159_R04C01
1.2
0.8
0.4
0.0
-0.4
-0.8
-1.2
MDM4
MYCN
GLI2
FGFR3/TACC3
PDGFRA
TERT
MYB
EGFR
CDK6
MET
KIAA1549/BRAF
FGFR1/TACC1
MYBL1
MYC
CDKN2A/B
PTCH1
PTEN
MGMT
CCND1
CCND2
CDK4
MDM2
RB1
TP53
NF1
PPM1D
C19MC
SMARCB1
NF2
chr1
chr2
chr3
chr4
chr5
chr6
chr7
chr8
chr9
chr10
chr11
chr12
chr13
chr14
chr15
chr16
chr17
chr18
chr19
chr20
chr21

#38

204958100051_R06C01

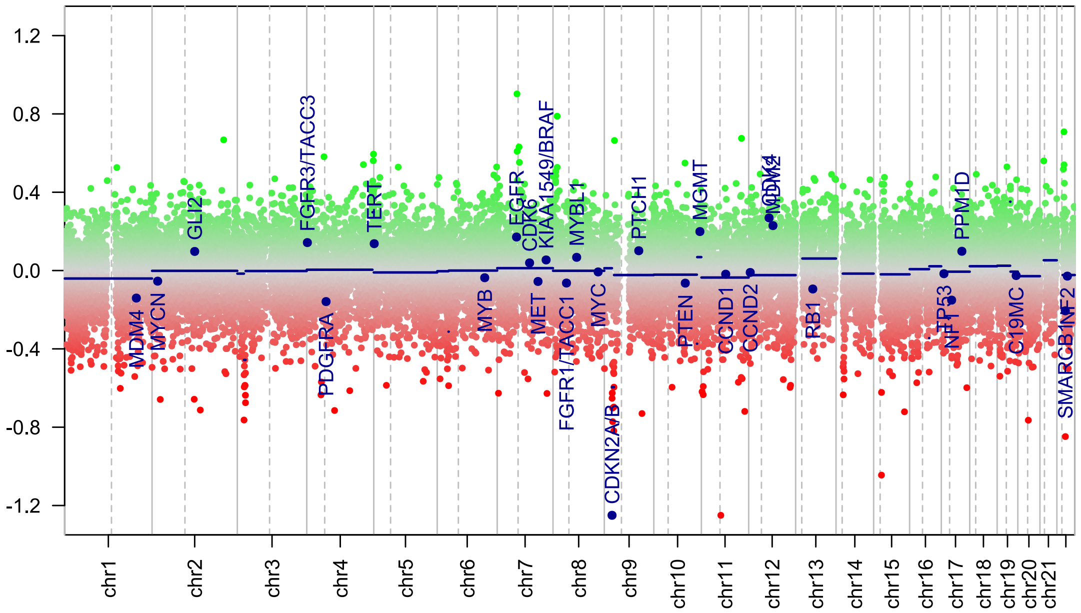

#39

206238140036_R01C01

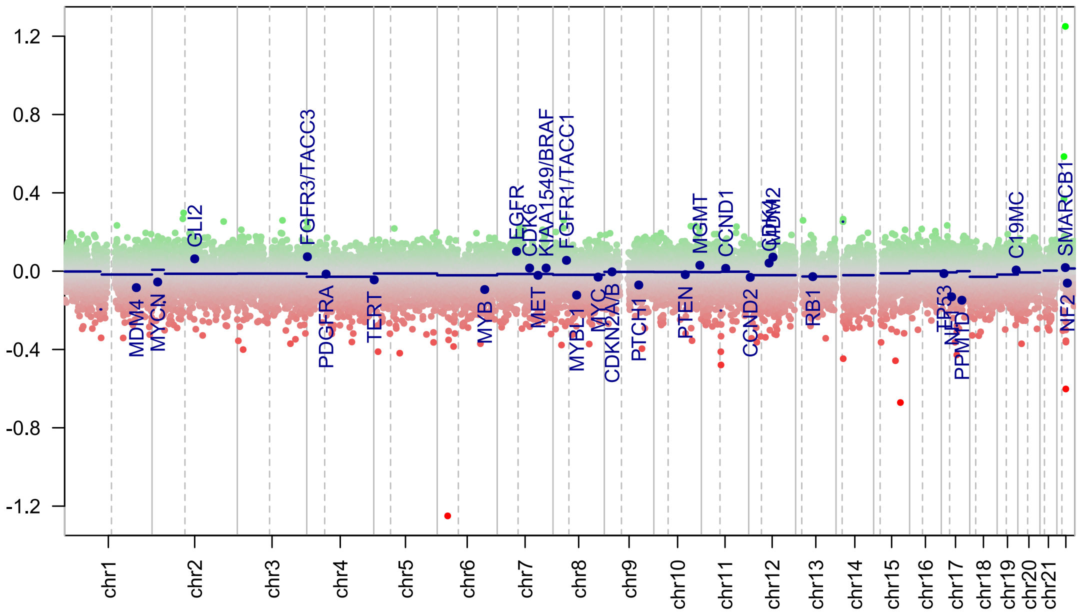

#40

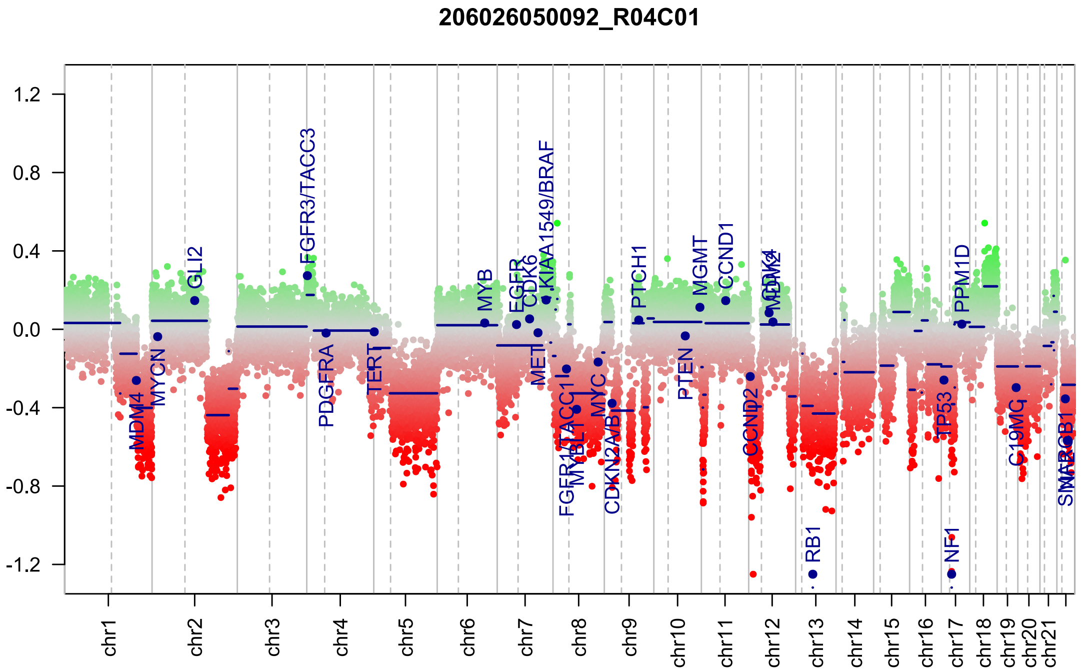

206026050092_R04C01
1.2
0.8
0.4
0.0
-0.4
-0.8
-1.2
chr1
chr2
chr3
chr4
chr5
chr6
chr7
chr8
chr9
chr10
chr11
chr12
chr13
chr14
chr15
chr16
chr17
chr18
chr19
chr20
chr21
MDM4
MYCN
GLI2
FGFR3/TACC3
PDGFRA
TERT
MYB
EGFR
CDK6
KIAA1549/BRAF
MET
PTCH1
PTEN
MGMT
CCND1
CCND2
RB1
TP53
NF1
PPM1D
C19MC

#41

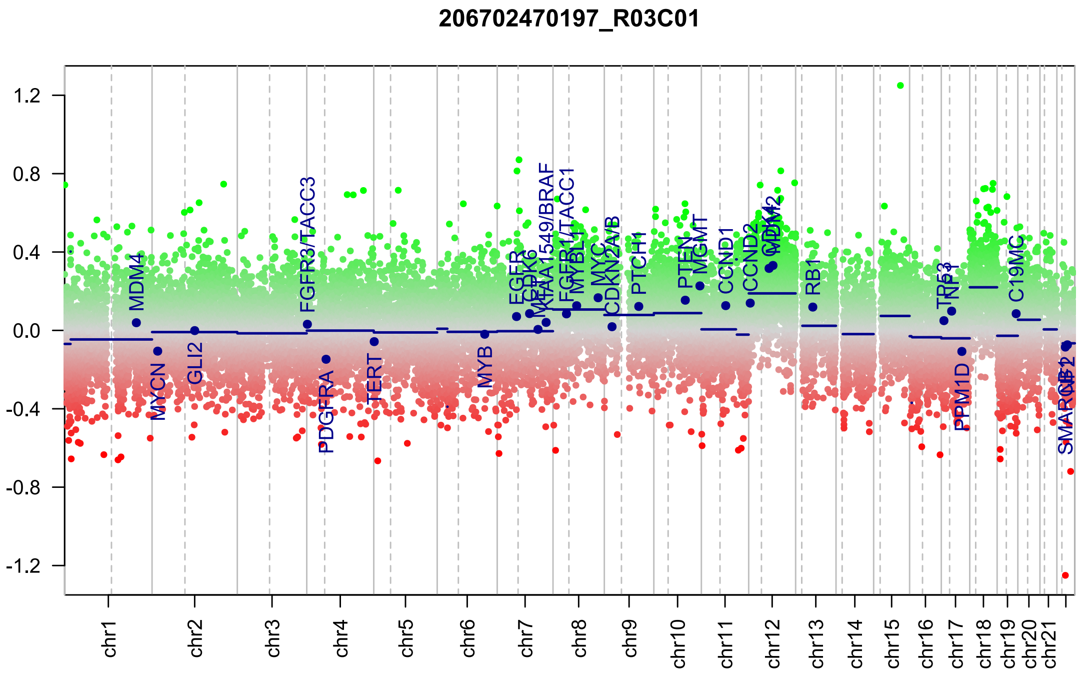

206702470197_R03C01
1.2
0.8
0.4
0.0
-0.4
-0.8
-1.2
chr1
chr2
chr3
chr4
chr5
chr6
chr7
chr8
chr9
chr10
chr11
chr12
chr13
chr14
chr15
chr16
chr17
chr18
chr19
chr20
chr21
MDM4
MYCN
GLI2
FGFR3/TACC3
PDGFRA
TERT
MYB
EGFR
KIAA1549/BRAF
FGFR1/TACC1
PTCH1
PTEN
MGMT
CCND1
CCND2
RB1
PPM1D
C19MC

#42

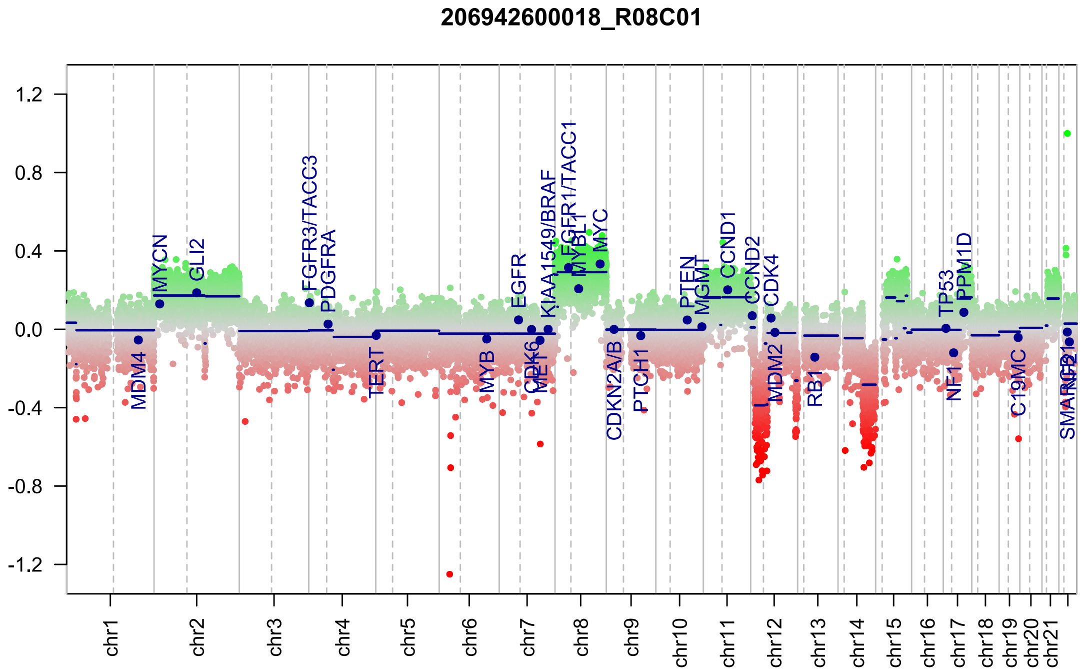

206942600018_R08C01
1.2
0.8
0.4
0.0
-0.4
-0.8
-1.2
MYCN
GLI2
MDM4
FGFR3/TACC3
PDGFRA
TERT
MYB
EGFR
CDK6
MET
KIAA1549/BRAF
FGFR1/TACC1
MYBL1
MYC
CDKN2A/B
PTCH1
PTEN
MGMT
CCND1
CCND2
CDK4
MDM2
RB1
TP53
PPM1D
NF1
C19MC
SMARCB1
NF2
chr1
chr2
chr3
chr4
chr5
chr6
chr7
chr8
chr9
chr10
chr11
chr12
chr13
chr14
chr15
chr16
chr17
chr18
chr19
chr20
chr21

Supplementary Figure 2

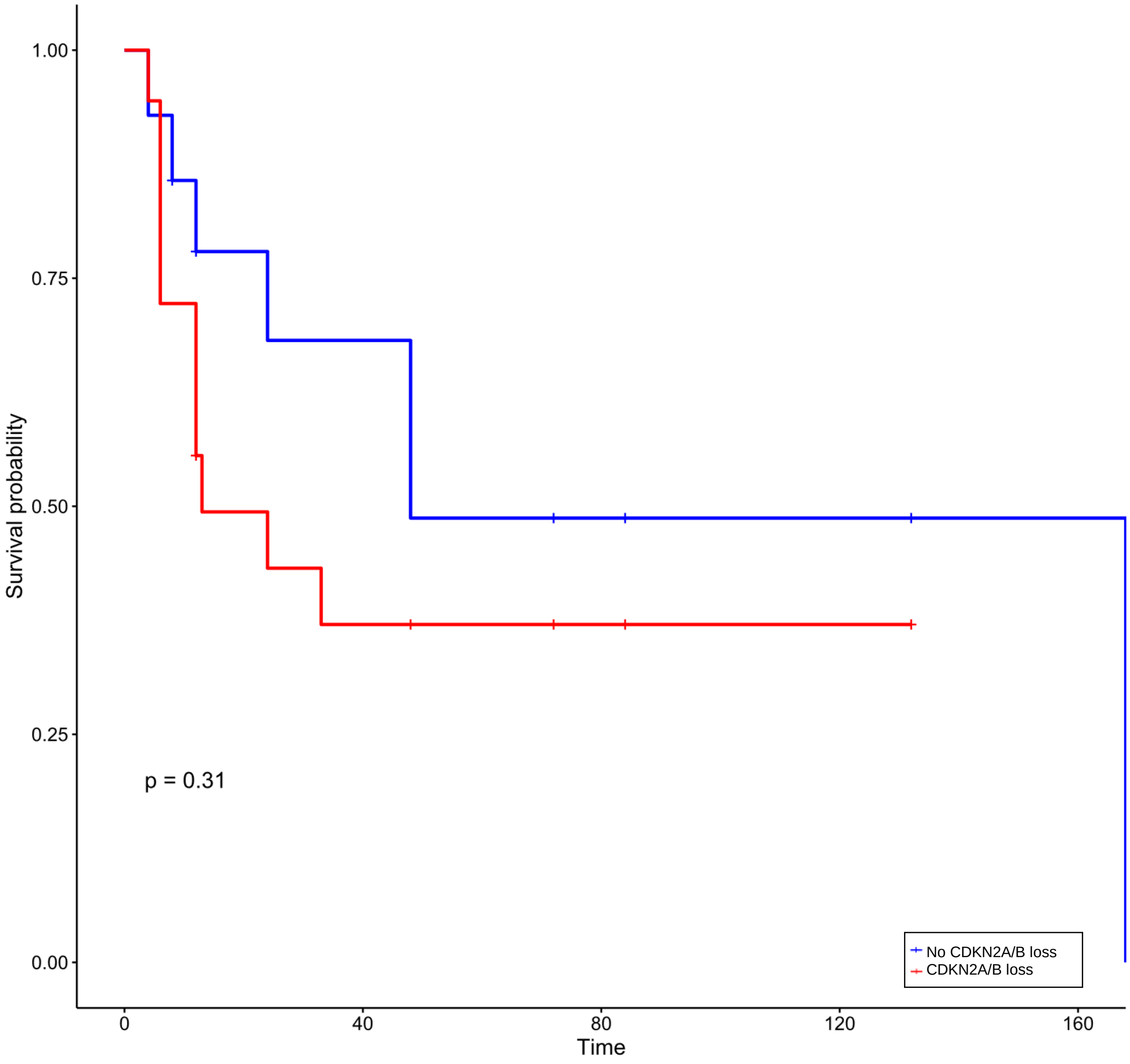

Supplement: Supplementary file 2 — Additional file 2: Supplementary Figure 1. Genome-wide CNV plots generated with conumee for each corresponding case number (#1-#42). Log2Ratio is represented on the Y axis, while the X axis represents the chromosomal position. Supplementary Figure 2. Kaplan-Meier curve representing the effect of CDKN2A/B loss (absent in blue, present in red) on all patients’ survival. [file 13148_2023_1621_MOESM2_ESM.pdf]
